# Expression of a constitutively active nitrate reductase variant in tobacco reduces tobacco‐specific nitrosamine accumulation in cured leaves and cigarette smoke

**DOI:** 10.1111/pbi.12510

**Published:** 2016-01-23

**Authors:** Jianli Lu, Leichen Zhang, Ramsey S. Lewis, Lucien Bovet, Simon Goepfert, Anne M. Jack, James D. Crutchfield, Huihua Ji, Ralph E. Dewey

**Affiliations:** ^1^ Department of Crop ScienceNorth Carolina State UniversityRaleighNCUSA; ^2^ Philip Morris International R&DPhilip Morris Products SANeuchatelSwitzerland; ^3^ Kentucky Research and Development CenterUniversity of KentuckyLexingtonKYUSA; ^4^ Department of Plant and Soil SciencesUniversity of KentuckyLexingtonKYUSA

**Keywords:** nitrate reductase, tobacco, tobacco‐specific nitrosamines, burley, nitrogen‐assimilation pathway, NNN, NNK

## Abstract

Burley tobaccos (*Nicotiana tabacum*) display a nitrogen‐use‐deficiency phenotype that is associated with the accumulation of high levels of nitrate within the leaf, a trait correlated with production of a class of compounds referred to as tobacco‐specific nitrosamines (TSNAs). Two TSNA species, 4‐(methylnitrosamino)‐1‐(3‐pyridyl)‐1‐butanone (NNK) and *N*‐nitrosonornicotine (NNN), have been shown to be strong carcinogens in numerous animal studies. We investigated the potential of molecular genetic strategies to lower nitrate levels in burley tobaccos by overexpressing genes encoding key enzymes of the nitrogen‐assimilation pathway. Of the various constructs tested, only the expression of a constitutively active nitrate reductase (NR) dramatically decreased free nitrate levels in the leaves. Field‐grown tobacco plants expressing this NR variant exhibited greatly reduced levels of TSNAs in both cured leaves and mainstream smoke of cigarettes made from these materials. Decreasing leaf nitrate levels via expression of a constitutively active NR enzyme represents an exceptionally promising means for reducing the production of NNN and NNK, two of the most well‐documented animal carcinogens found in tobacco products.

## Introduction

Cured leaves of different tobacco market types (flue‐cured, burley and Oriental) are typically blended together in the production of cigarettes. Of these market types, burley tobaccos are unique in that they display a chlorophyll‐deficient phenotype and are greatly impaired in their nitrogen‐use and nitrogen utilization efficiencies (N‐USE and N‐UTL, respectively) (Henika, 1932; Lewis *et al*., 2012; Stines and Mann, 1960). As a result, burley tobaccos require much higher levels of N‐fertilization than other tobacco types to achieve comparable yields. Genetic studies have determined that loci designated *yb1* and *yb2* account for the major differences observed between flue‐cured and burley tobaccos (Legg *et al*., 1977). Although the function of *yb1* and *yb2* is unknown, it has been established that the genetic differences are manifested within the leaf, as opposed to root tissue (Crafts‐Brandner *et al*., 1987a,b).

As a likely consequence of their deficient N‐UTL phenotype, burley plants also accumulate higher levels of free nitrate in their leaves than most other tobacco types (Burton *et al*., 1994), a trait that is also attributable to the *yb1* and *yb2* loci (Lewis *et al*., 2012). High levels of nitrate are associated with the formation of compounds referred to as tobacco‐specific nitrosamines (TSNAs) (Bush *et al*., 2001; Fischer *et al*., 1989; Lewis *et al*., 2012). TSNAs are produced via nitrosation of naturally occurring tobacco alkaloids in processes that predominantly take place during the curing of tobacco leaves, although additional formation can occur in the subsequent storage and processing of the leaf, and in some circumstances via pyrosynthesis during combustion. Two of the TSNAs found in the cured leaf, *N*‐nitrosonornicotine (NNN) and 4‐(methylnitrosamino)‐1‐(3‐pyridyl)‐1‐butanone (NNK), derived from nitrosation of nornicotine and nicotine, respectively, are classified as Group I carcinogens (the highest designation) by the International Agency for Research on Cancer (2007). Given that NNN and NNK are among the most potent animal carcinogens found in tobacco products (Hecht, 1998, 2008), there is great interest in reducing their levels in these products.

Genetic strategies to lower TSNA content have focused on targeting either (i) the alkaloid precursor(s) or (ii) the nitrosating agent(s) involved. The effectiveness of the former strategy is well exemplified by the substantial reductions in NNN that were observed through the down‐regulation of the gene family responsible for the synthesis of its alkaloid precursor nornicotine (Dewey and Xie, 2013; Lewis *et al*., 2008). In air‐cured tobaccos, there is a general consensus that nitrite is the nitrosating agent directly responsible for TSNA formation (Bush *et al*., 2001). Due to its cellular toxicity, however, endogenous nitrite levels are typically very low in plant tissues. Instead, it is believed that the great majority of the nitrite involved in TSNA formation is derived from the nitrate reductase (NR) activity of microbes residing on the leaf surface that convert a portion of the leaf nitrate pools to nitrite as cellular membranes and organelles degrade during the 6‐ to 10‐week curing period (Bush *et al*., 2001). Thus, if the amount of free nitrate stored within the leaf could be reduced, TSNA formation may be decreased as well.

**Figure 1:**
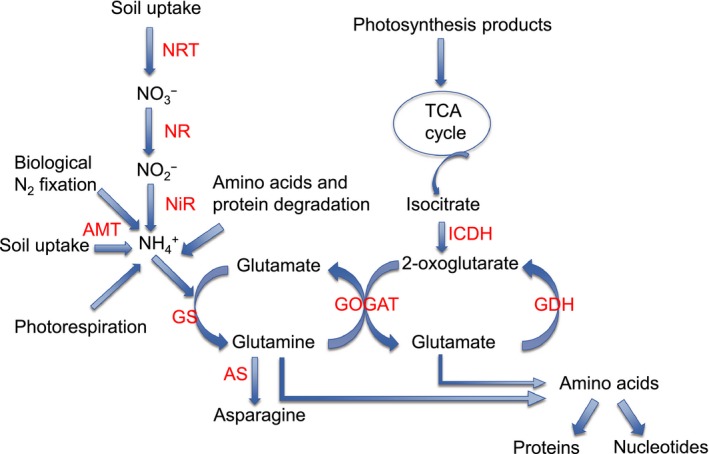
Nitrogen‐assimilation pathway in higher plants. Inorganic nitrogen in the form of nitrate or ammonia becomes incorporated into amino acids and other organic molecules as depicted. The specific steps shown include: nitrate transporters (NRT), nitrate reductase (NR), nitrite reductase (NiR), ammonium transporters (AMT), glutamine synthetase (GS), glutamate synthase (GOGAT), asparagine synthetase (AS), glutamate dehydrogenase (GDH), and isocitrate dehydrogenase (ICDH).

As burley tobaccos show deficiencies in N‐USE and N‐UTL and display a higher nitrate accumulation phenotype compared to other tobacco types, it is possible that one or more steps of the N‐assimilation pathway is impaired. A simplified diagram of the N‐assimilation pathway in plants is shown in Figure 1. Because of their central roles within the primary N‐assimilation pathway, researchers have frequently manipulated the expression of genes encoding the enzymes NR, glutamate synthase (GOGAT) and glutamine synthetase (GS) in attempts to alter the flux of metabolites through this pathway (Good *et al*., 2004; Harrison *et al*., 2000). Another potentially important step in this process is catalysed by isocitrate dehydrogenase (ICDH), the enzyme that is believed to be responsible for providing the 2‐oxoglutarate carbon skeleton required by GOGAT to produce glutamate (Hodges *et al*., 2003).

Studies of plant NR genes and their encoded enzymes have shown that this step of the N‐assimilation pathway is highly regulated at both the transcriptional and post‐transcriptional levels. Because of post‐translational regulatory mechanisms, the overexpression of NR genes in transgenic plants typically leads to only modest increases in NR activity in the cell. Post‐translational controls of NR were at least partially overcome when a truncated tobacco NR gene was expressed in a NR‐deficient mutant of *N. plumbaginifolia* (Nussaume *et al*., 1995). Specifically, a 56 amino acid deletion near the N‐terminus enabled the enzyme to be equally active in both the light and dark, compared to the wild‐type (WT) enzyme that becomes inactivated during dark cycles. Another distinct mutation of the tobacco NR gene that resulted in the elimination of post‐translational regulatory controls involved the substitution of a serine (Ser) residue at position 523 with an aspartate (Asp) (Lillo *et al*., 2003). The S523D‐NR enzyme no longer served as a substrate for the protein kinases that normally inactivate this enzyme during the dark via phosphorylation of Ser 523. Although research using the S523D‐NR variant demonstrated that its overexpression could significantly lead to enhanced NR activity and substantial changes in N‐assimilation metabolites, such as nitrate, ammonia, and amino acid levels in transgenic *N. plumbaginifolia*, no differences in overall growth phenotype were observed when the plants were grown under normal conditions (Lea *et al*., 2006; Lillo *et al*., 2003). [As an aside, the papers by Lillo *et al*. (2003) and Lea *et al*. (2006) mistakenly refer to amino acid position 521 as the location of the Ser to Asp mutation. From the original description of this pivotal regulatory site in the NR enzyme from spinach, however, it is clear that the Ser residue at position 523 (not 521) represents the analogous site in tobacco NR enzymes (see Fig. 8 of Bachmann *et al*., 1996)].

Ammonia [which will be used to collectively refer to both ammonia (NH_3_) and ammonium ions (${\mathrm{NH}}_{4}^{+}$)] in plant tissues is not only derived from the reduction of nitrite, but can also be taken up directly from the soil. Through the GS/GOGAT cycle, ammonia is subsequently incorporated into amino acids. Two distinct isoforms of GS have been characterized in plants, one of which is a cytosolic isoform (GS1) and the other one being plastid localized (GS2) (McNally *et al*., 1983). Of the two isoforms, GS1 plays the most important role in the primary assimilation of nitrate and/or ammonia taken up from soil (Bernard and Habash, 2009; Oaks and Hirel, 1985). Similarly, two isoforms of GOGAT have been characterized in plants, a ferredoxin‐dependent enzyme (Fd‐GOGAT) and a NADH‐dependent isoform (NADH‐GOGAT). Overexpression of an alfalfa NADH‐GOGAT gene in tobacco modestly increased total C and N concentrations and shoot dry weight (Chichkova *et al*., 2001). The 2‐oxoglutarate that serves as the carbon skeleton substrate of GOGAT is likely provided by the ICDH‐catalysed oxidative decarboxylation of isocitrate. Although the role of ICDH is presumed to be important to the GS/GOGAT cycle, we are aware of no reports documenting the effects of ICDH gene overexpression in tobacco, or any other plant.

The purpose of this study was to: (i) determine whether overexpression of a GS1, NADH‐GOGAT, ICDH or constitutively active NR enzyme could overcome the unfavourable effects of the *yb1* and *yb2* loci that give rise to the high nitrate phenotype characteristic of burley tobaccos and (ii) establish the degree by which TSNA levels may be reduced in plants where nitrate concentrations may have been successfully decreased. Here, we demonstrate that ectopic expression of a the S523D‐NR variant facilitates dramatic reductions in the nitrate content of the leaf, a phenotype that is accompanied by substantial reductions in the TSNA content of the cured leaf as well as the corresponding mainstream cigarette smoke.

## Results

### Overexpression of N‐assimilation genes in burley tobacco

To determine whether the overexpression of GS1, NADH‐GOGAT, ICDH or a constitutively active NR enzyme could help alleviate the high nitrate accumulating phenotype attributed to the *yb1* and *yb2* loci, the following five gene constructs were individually overexpressed in burley tobacco line DH98‐325‐6 #775 (Lewis *et al*., 2010): S523D‐NR, a tobacco *Nia2* cDNA (Vaucheret *et al*., 1989) containing a Ser to Asp substitution mutation introduced at codon 523 (Lillo *et al*., 2003); tr‐NR, a cDNA containing a 56 amino acid truncation near the N‐terminus of *Nia2* (Nussaume *et al*., 1995); GS1, a full‐length cDNA of the tobacco *Gln1‐3* gene (Dubois *et al*., 1996); NADH‐GOGAT, the full‐length cDNA of the *GLT1* gene of *Arabidopsis thaliana* (Lancien *et al*., 2002); and ICDH, a full‐length cDNA of a tobacco cytosolic isocitrate dehydrogenase (GenBank accession #X77944).

Seed was harvested from the three T0 lines of each construct that displayed the highest levels of transgene transcript accumulation as analysed by semi‐quantitative RT‐PCR (data not shown). Semi‐quantitative RT‐PCR was also conducted on progeny of each selected high expressing T0 plant to test whether the T1 generation plants that inherited the transgene(s) continued to express it at a high level. All plants used in subsequent studies were T2 plants taken from T1 seed lots where all progeny that tested positive for the transgene (via molecular genotyping) continued to express the transgene at a high level. T2 segregants that no longer possessed a transgene were used as the source for WT (null segregant) controls.

### Analysis of transgenic plants in a controlled environment

**Figure 2:**
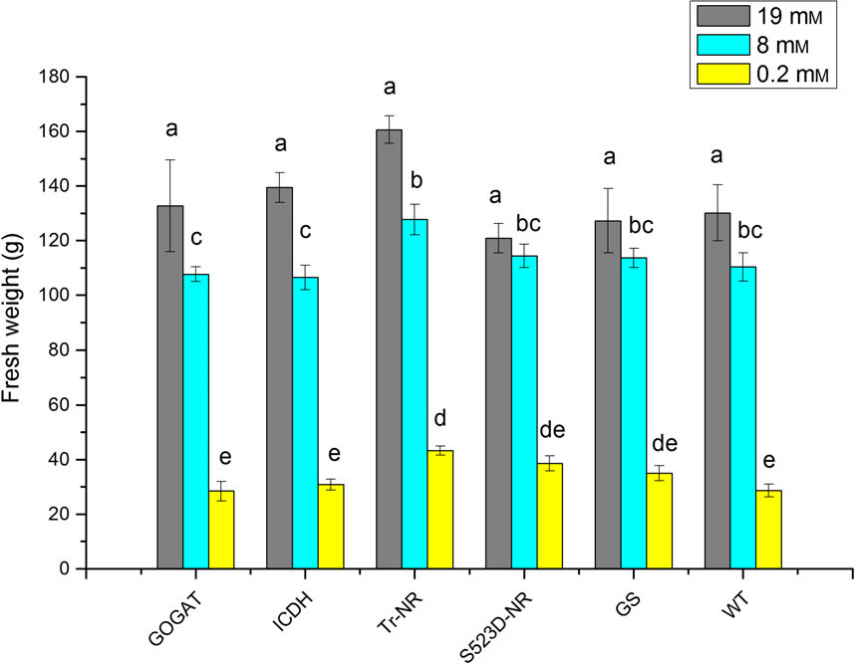
Fresh weights of WT plants and 35S:tr‐NR, 35S:S523D‐NR, 35S:GS1, 35S:GOGAT and 35S:ICDH transgenic lines grown under three levels of N‐fertilization. Values shown represent the nontransformed means ± standard errors of 4–6 plants for each genotype. Statistical tests were performed on transformed data (natural logarithmic transformation). Within each nitrate treatment level, means sharing the same letter are not significantly different from each other (*P *<* *0.05); the letter ‘a’ only applies to plants treated with 19 mm nitrate, letters ‘b’ and ‘c’ apply only to plants watered with the 8 mm nitrate media, and ‘d’ and ‘e’ only apply to plants treated with 0.2 mm nitrate.

To examine the effects of N‐assimilation pathway gene overexpression in tobacco plants grown under varying conditions of nitrate availability, three sets of 8‐week‐old T2 plants for each transgene genotype (35S:tr‐NR, 35S:S523D‐NR, 35S:GS1, 35S:GOGAT and 35S:ICDH) and WT controls were grown for an additional 16 days in a controlled environment growth chamber under nitrogen regimes containing 0.2, 8 or 19 mm nitrate. As expected, WT tobacco plants watered with media containing 0.2 mm nitrate were chlorotic and displayed the least amount of growth, while plants provided with 19 mm nitrate were the largest and darkest green. As a typical example, pictures of the WT and 35S:tr‐NR transgenic plants at the end of the 16‐day treatment period are shown in Figure S1. No significant differences in fresh weight were observed among the various transgenic genotypes versus WT controls for plants given the 8 and 19 mm nitrate treatments (Figure 2). At the very low nitrate treatment, however, the fresh weight of plants expressing the 35S:tr‐NR construct was statistically significantly greater than WT.

The chlorophyll deficiencies of burley tobaccos are clearly distinguishable by their lighter green appearance (Henika, 1932; Stines and Mann, 1960). If any of the transgenes overexpressed in this study were capable of compensating for the *yb1* and/or *yb2* mutations, one would expect to see an increase in the chlorophyll content of these plants. Measurements of chlorophyll a, b and a + b showed a very strong treatment effect across the entire study with chlorophyll content being positively correlated with increased nitrate fertilization (Figure S2 and Table S1); however, no significant differences in chlorophyll concentrations were observed between any transgene genotype and the WT controls within any of the three nitrate treatments. These results suggest that the overexpression of the N‐assimilation pathway associated genes included in this study did not complement, or otherwise functionally bypass, the *yb1* and/or *yb2* mutant loci that define the burley phenotype.

**Figure 3:**
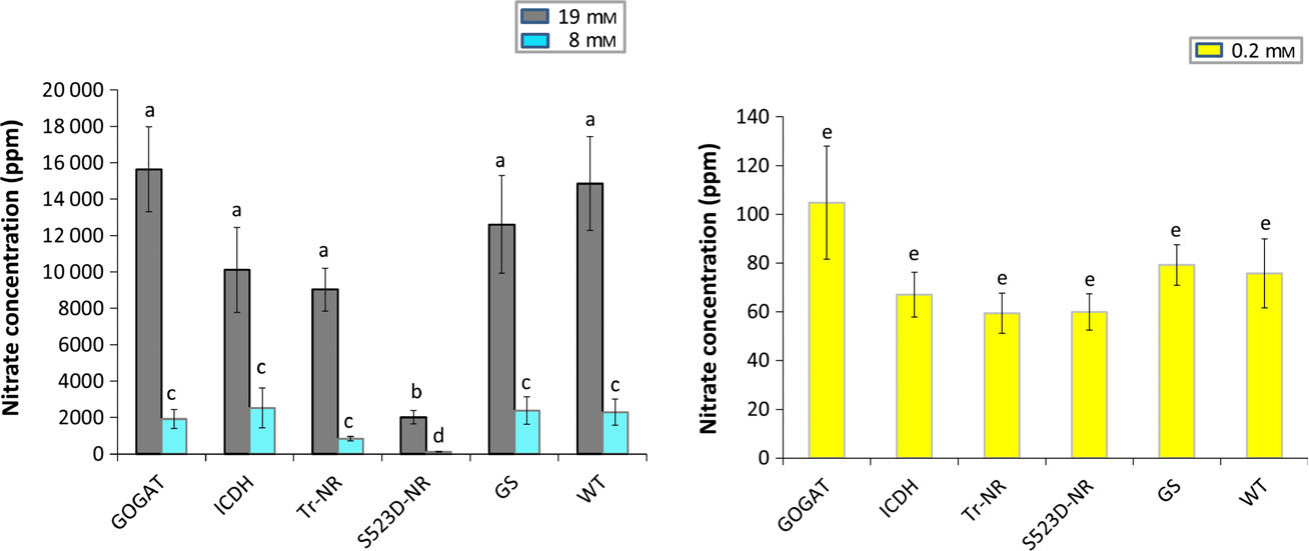
Total nitrate content in leaves of WT plants and 35S:tr‐NR, 35S:S523D‐NR, 35S:GS1, 35S:GOGAT and 35S:ICDH transgenic lines grown under three levels of N‐fertilization. Data from plants grown using the 19 mm ${\mathrm{NO}}_{3}^{-}$ and 8 mm ${\mathrm{NO}}_{3}^{-}$ treatments are shown on the left; results from the 0.2 mm ${\mathrm{NO}}_{3}^{-}$ treatment are shown on the right. Values shown represent the nontransformed means ± standard errors of 4–6 plants for each genotype. Statistical tests were performed on transformed data (natural logarithmic transformation). Within each nitrate treatment level, means sharing the same letter are not significantly different (*P *<* *0.05) as explained in Figure 2.

The nitrate and ammonia concentrations for the various genotypes at each nitrate fertilization level were measured (Table S1). At the 0.2 mm nitrate treatment level, all plants displayed exceptionally low nitrate accumulation, and no significant differences were observed between any transgene genotype and the WT controls (Figure 3). Under the 8 mm and 19 mm nitrate treatments, the average concentration of nitrate measured in the 35S:tr‐NR plants was 37% and 60% of WT controls, respectively. These differences were not statistically significant, however, using the stringent Ryan–Einot–Gabriel–Welsch (REGW) multiple comparison test that we employed for data analysis. Plants containing the 35S:S523D‐NR construct, however, accumulated only about 5% of the amount of nitrate present in the control plants when fertilized with 8 mm nitrate (Figure 3). At the 19 mm N‐fertilization treatment, WT plants averaged approximately 14 900 ppm nitrate, compared with only ~2000 ppm observed in the 35S:S523D‐NR plants, a near eightfold reduction.

Although ammonia levels did not vary as much as nitrate, the 35S:S523D‐NR plants accumulated approximately twice as much ammonia as the control plants at the 19 mm nitrate treatment level (Figure S3). At the low and medium N treatment levels, however, no given transgenic genotype was statistically different in its average ammonia content from the WT controls, or any other transgenic genotype.

As the N‐assimilation pathway feeds directly into amino acid biosynthesis, we sought to establish whether concentrations of free amino acids were altered in the transgenic plants. Because only minimal differences in nitrate and ammonia levels were observed in plants fertilized with the 0.2 mm nitrate media (Figures 3 and S3), the quantification of free amino acids was only conducted on the plants treated with 8 and 19 mm nitrate. The most striking observation was the great increase in total free amino acids in plants harbouring the 35S:S523D‐NR construct fertilized with 19 mm nitrate (Figure S4). Tobacco plants expressing this construct accumulated on average 3.7 times more total free amino acids than WT plants. Although virtually every amino acid was higher in the 35S:S523D‐NR group at the high N treatment, levels of Gln, Asn and Arg were particularly elevated, showing increases of 8.5‐, 5.5‐ and 5.3‐fold, respectively (Figure S5). When grown using the 8 mm nitrate nutrient solution, 35S:S523D‐NR plants accumulated Gln and Asn at significantly higher levels than the control (twofold and 3.4‐fold, respectively), yet the total amino acid concentrations between the two lines were nearly identical, due to mean decreases in the levels of several other amino acids in the 35S:S523D‐NR plants (Table S2). As a whole, the amino acid profile of plants expressing the other NR gene variant, 35S:tr‐NR, was similar to WT plants. Our observations of the 35S:S523D‐NR construct being more effective than 35S:tr‐NR in lowering the nitrate levels and altering the amino acid profile of the leaf are similar to that reported when these constructs were expressed in *N. plumbaginifolia* (Lea *et al*., 2006). The overall impact of reducing the levels of stored nitrate in 35S:S523D‐NR plants with respect to free amino acids and ammonia across the two fertilization regimes is visually summarized as a heat map in Figure S6.

Glutamine is the reaction product of the GS1 enzyme. Interestingly, Gln was not increased in plants overexpressing the GS1 gene (Figure S5 and Table S2). This is in contrast to the study of Fuentes *et al*. (2001) where a twofold increase in Gln was observed when an alfalfa GS cDNA was overexpressed in tobacco plants and fertilized with a high nitrate solution. Likewise, the levels of Glu were not significantly increased in transgenic plants overexpressing the gene encoding the GOGAT enzyme that is directly responsible for its synthesis (Figure 1). At the 8 mm nitrate fertilization level, plants overexpressing ICDH displayed lower amino acids in total (Figure S4), and lower amounts of Glu specifically (Figure S5) than any of the other genotypes.

### Analysis of transgenic plants grown under field conditions

The results of the growth chamber experiments revealed that the 35S:S523D‐NR construct was able to mediate dramatic reductions in leaf nitrate content. The mean nitrate content of the 35S:tr‐NR plants was the second lowest in this study. To determine the effects of the 35S:S523D‐NR and 35S:tr‐NR constructs on cured leaf nitrate content in a cultivated field environment, T2 plants originating from the same seed lots used in the growth chamber‐based experiment were grown in two field locations, topped and harvested using standard cultivation practices. In addition, because the consequences of excess free nitrate in the leaf with respect to TSNA formation is primarily manifested during leaf senescence and curing, we speculated that it may be advantageous to place the constitutively activated NR constructs under the transcriptional control of a strong senescence‐specific promoter. The *CYP82E4* nicotine demethylase gene specifically mediates a high level of gene expression during senescence and air‐curing (Chakrabarti *et al*., 2008). Constructs placing the S523D‐NR and tr‐NR cDNAs under the control of the *CYP82E4* (E4) promoter and *nos* terminator were generated and transformed into burley line DH98‐325‐6 #775. As a control representing the overexpression of an N‐assimilation pathway gene that does not influence leaf nitrate content, 35S:GOGAT and E4:GOGAT transgenic plants were also included in the field study. The specific T2 populations tested in the field possessing the E4 promoter‐driven constructs came from seed lots of T1 plants that expressed the transgenes at a high level when treated with the senescence‐inducing compound ethephon (Chakrabarti *et al*., 2008). Similar to the growth chamber studies, null segregants from T1 plants that no longer possessed a transgene were used as the source for WT controls.

Following standard production practices for burley tobaccos, mature, stalk‐harvested plants were air‐cured for approximately 10 weeks. At the end of this period, the third and fourth leaves from the top of each plant were harvested, stripped of the mid‐rib and dried to completeness. Nitrate analysis of these samples demonstrated that leaves of the 35S:S523D‐NR genotype accumulated far less free nitrate than any other genotypic group, and only ~4% of that observed in WT plants (Table 1). Cured leaf samples of genotypes containing tr‐NR constructs displayed more modest, yet statistically significant reductions in nitrate content compared to WT plants, ranging from 70% to 77% of the nitrate concentration observed in the WT group.

**Table 1:** The effect of nitrate‐related gene constructs on ${\mathrm{NO}}_{3}^{-}$ content in the upper leaves of field‐grown T2 generation transgenic burley plants after 10 weeks of air‐curing

Genotype	Mean (ppm DW)	*n*	REGW grouping
Wild type	2806.1	79	A
E4:GOGAT	2493.3	63	BA
35S:GOGAT	2451.4	79	BA
E4:tr‐NR	2149.9	76	BC
E4:S523D‐NR	2035.1	75	C
35S:tr‐NR	1974.7	70	C
35S:S523D‐NR	107.4	60	D

Analysis was conducted on plants grown in two locations (no significant location effect detected). Means with the same letter are not significantly different at α = 0.05 by the REGW method.

Given that nitrite is ultimately the compound believed to be responsible for TSNA formation during air‐curing, it is important that genetic modifications that lead to reductions in nitrate pools do not result in an increase in the levels of nitrite within the cured leaf. As shown in Table S3, nitrite levels among the various genotypes were similar. Although the numeric mean of the 35S:S523D‐NR group was the lowest of all the genotypes, it was not statistically different from the WT controls.

Thirty‐three randomly chosen cured leaf samples from each of the WT, 35S:S523D‐NR and 35S:GOGAT genotypic groups were selected for analysis of alkaloids, free amino acids, ammonia and TSNAs. The results of the alkaloid analysis (Table 2) indicated no significant differences in total alkaloid content, or any individual alkaloid (nicotine, nornicotine, anabasine and anatabine) among the three genotypes. Although the levels of certain amino acid species (e.g. Ala and Asp) were significantly higher in the 35S:S523S‐NR individuals than in the 35S:GOGAT and WT controls, total amino acid content was not considered significantly different at an α=0.05 threshold (Table S4). Ammonia levels were likewise not deemed to be statistically different among the three genotypes (Table S4).

**Table 2:** TSNA and alkaloid content in cured leaves of field‐grown WT, 35S:GOGAT and 35S:S523D‐NR plants

	WT	35S:GOGAT	35S:S523D‐NR
Total TSNA (ng/g)	709	649	162
	A	A	B
NNN (ng/g)	287	288	31
	A	A	B
NAT (ng/g)	293	257	83
	A	A	B
NAB (ng/g)	17	14	0.3
	A	A	B
NNK (ng/g)	111	91	48
	A	A	B
Total alkaloid (%)	2.6	2.8	2.6
	A	A	A
Nicotine (%)	2.5	2.6	2.4
	A	A	A
Nornicotine (%)	0.058	0.063	0.058
	A	A	A
Anabasine (%)	0.011	0.011	0.012
	A	A	A
Anatabine (%)	0.075	0.075	0.091
	A	A	A

Means with the same letter are not significantly different at α=0.01. Alkaloid measurements represent % dry weight. Means are grouped according to the REGW method, *n *=* *33.

Major differences in TSNA content were apparent in the low nitrate accumulating 35S:S523D‐NR leaf samples. A 90% reduction in the levels of NNN was observed in the 35S:S523D‐NR group compared to WT controls. *N*‐nitrosoanatabine (NAT) and NNK accumulation were reduced by 72.5% and 55%, respectively, in 35S:S523D‐NR versus WT plants, and the *N*‐nitrosoanabasine (NAB) content in 35S:S523D‐NR plants was nearly undetectable (Table 2). Cumulatively, the total reduction in TSNA content in the upper leaf samples of the 35S:S523D‐NR genotype compared with WT was 77.5%. No statistically significant differences were observed for any individual TSNA or total TSNA content in WT plants versus the 35S:GOGAT plants. These results clearly demonstrate that 35S promoter‐driven expression of the S523D‐NR construct leads to substantial reductions in the TSNA content of air‐cured tobacco leaves, a phenomenon most reasonably attributable to its ability to dramatically reduce the levels of free nitrate within the leaf.

### Analysis of the cut filler and cigarette smoke

In combustible tobacco products such as cigarettes, it is the level of toxicants found in the smoke that ultimately constitutes the exposure. The TSNA content of cigarette smoke is dependent on: (i) the amount of pre‐existing TSNAs in the cut filler tobacco; (ii) the efficiency of transfer of the pre‐existing TSNAs from the tobacco to the smoke; (iii) the extent of the destruction of pre‐existing TSNAs via pyrolysis; and (iv) the extent of formation of new TSNAs via pyrosynthesis. To determine the impact of the reduced nitrate phenotype on the prevalence of TSNAs in mainstream smoke, cigarettes were made from pooled samples of the remaining cured leaf materials (predominantly lower stalk position leaves) of the field‐grown 35S:S523‐NR plants and the WT controls.

**Figure 4:**
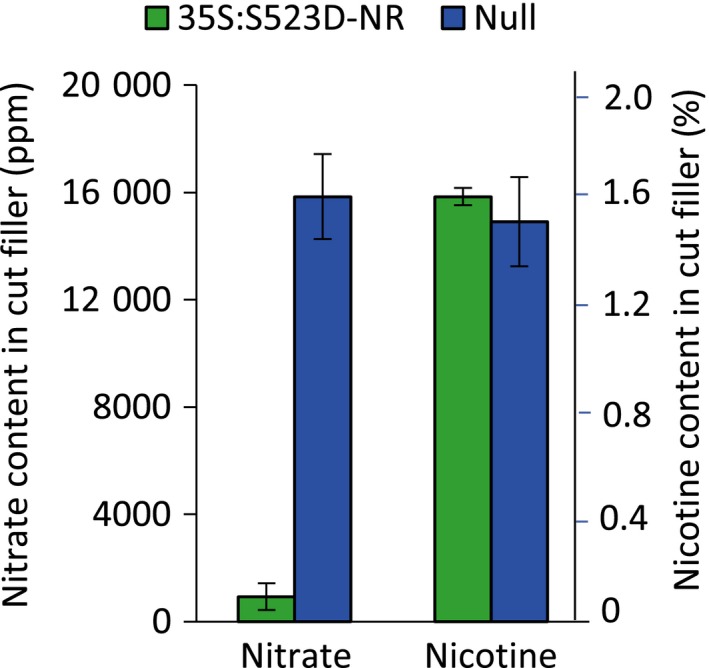
Average nitrate and nicotine content in cut tobacco filler derived from the lamina of WT and 35S:S523D‐NR genotypes. All plant materials of each genotype were pooled according to location (about 30 plants each from Clayton and Rocky Mount for 35S:S523D‐NR; about 40 plants from each location for WT). Data shown represent the average and standard error across both locations.

Cut filler for cigarette production was generated by finely shredding the cured leaf lamina. Because the relative concentrations of nitrate, nicotine and TSNAs can differ substantially according to stalk position, it was important to independently determine the levels of these compounds as represented in the cut filler in order to accurately interpret the corresponding smoke data. As shown in Figure 4, the nitrate concentration averaged 15 800 ppm in the cut filler from WT leaf lamina. In contrast, in cut filler generated from 35S:S523D‐NR plants, nitrate levels averaged 930 ppm, a value approximately 5.9% of that observed in the WT filler, and consistent with the ratio observed between these two genotypes in cured, upper stalk leaves (Table 1). Nicotine content in the cut filler of WT and 35S:S523D‐NR plants averaged 1.6% and 1.7% dry weight, respectively (Figure 4). The higher mean levels of nitrate and lower mean concentration of nicotine in the cut filler tobacco (lower leaf) versus the upper leaf samples (compare Figure 4 with Tables 1 and 2) is consistent with the results of previous studies that have documented the differences in the levels of these constituents in tobacco according to leaf position (Burton *et al*., 1994; Djordjevic *et al*., 1989; Hamilton *et al*., 1982).

**Figure 5:**
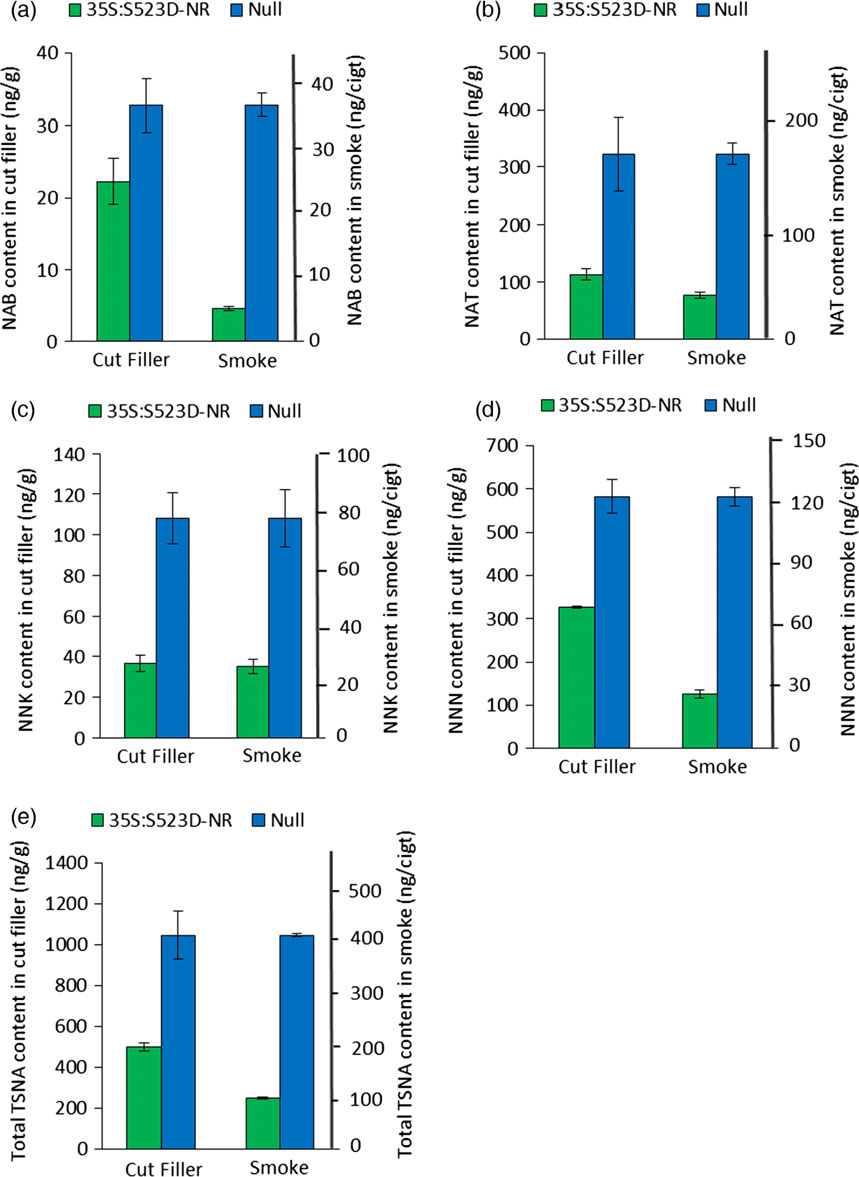
Average content of NAB (a), NAT (b), NNK (c), NNN (d) and total TSNAs (e) in the cut filler and smoke of cigarettes made from the lamina of WT and 35S:S523D‐NR leaves. Leaf materials of each genotype were pooled according to each of two locations as described in Fig. 4. Data shown represent the combined means and standard error of two replications per location for the TSNA content of the cut filler, and three replications per location for TSNA content in mainstream smoke.

Tobacco‐specific nitrosamine data for the cut filler tobacco are shown in Figure 5. Similar to the observations using cured upper leaves (Table 2), major reductions were observed in each of the four TSNA species assayed using cut filler from low nitrate plants, although some differences were observed in the relative extent of the respective reductions. Cut filler derived from 35S:S523D‐NR plants contained 44%, 64%, 65% and 32% less NNN, NNK, NAT and NAB, respectively, than filler made from WT control plants, with a total observed TSNA reduction of 52%. For both genotypes, TSNA levels were somewhat higher in the cut filler materials compared to the values measured in their corresponding cured upper leaves (compare Table 2 and Figure 4), a result consistent with previous observations that TSNA accumulation is higher in leaves from lower stalk positions (Burton *et al*., 1994).

Tobacco‐specific nitrosamine data from mainstream smoke derived from cigarettes made with the WT and 35S:S523D‐NR cut filler are also shown in Figure 5. The amount of total TSNA reduction attributable to the low nitrate phenotype was even greater in the mainstream smoke than that observed in the corresponding cut filler. The mainstream smoke of 35S:S523D‐NR cigarettes showed a 76% reduction in total TSNA content compared to WT cigarettes, as opposed to the 52% reduction observed in their cut filler tobacco (Figure 5e). The differences in the extent of reduction in TSNA content between 35S:S523D‐NR and WT cut filler tobaccos versus their corresponding mainstream smoke were most pronounced for NNN and NAB. The NNN content of unburned filler tobacco from 35S:S523D‐NR plants was 44% less than the WT filler, yet the differential in the mainstream smoke increased to 78% (Figure 5d). Likewise, NAB levels of 35S:S523D‐NR plants were 32% lower than WT in the filler, compared to an 86% reduction in the mainstream smoke (Figure 5a). Of the four TSNA species measured, only NNK failed to show a statistically significant differential in its relative reduction in the cut filler versus mainstream smoke when the results of WT and 35S:S523D‐NR cigarettes were compared. In both the cut filler and mainstream smoke, NNK was reduced by approximately 67% in cigarettes made using the low nitrate 35S:S523D‐NR tobacco (Figure 5c).

## Discussion

*N*‐nitrosonornicotine and NNK are two of the most thoroughly investigated toxicants found in tobacco products. NNK has been reported as likely being a major determinant in lung cancers, while NNN has been associated with cancers of the oesophagus, and oral and nasal cavities (Hecht, 2003, 2008). At the level of tobacco genetics, considerable progress has been made towards developing strategies that effectively reduce NNN levels in the cured leaf. Inhibition of the nicotine demethylase gene activities responsible for the production of the NNN precursor nornicotine (Siminszky *et al*., 2005) has been shown to reduce the levels of nornicotine and NNN by as much sixfold compared to commercial burley varieties (Lewis *et al*., 2008, 2010). Given that the strategy presented in this report is mechanistically distinct from the aforementioned, combining the low nitrate mediating 35S:S523D‐NR technology with the mutant nicotine demethylase technologies will likely result in even greater reductions in NNN than could be achieved by either approach individually.

Overexpression of the S523D‐NR construct reported here resulted in dramatic reductions in nitrate levels within a burley background. Burley tobaccos were specifically chosen for this study because they generally accumulate higher levels of nitrate than other tobacco types. Dark tobaccos are another tobacco type known for accumulating high nitrate levels. Given that the mechanism by which the constitutively active NR enzyme functions to deplete the nitrate pool would not be expected to differ among the various tobacco classes, one particularly promising application would be the introduction of the 35S:S523D‐NR construct into dark tobacco varieties. Dark tobaccos are primarily used for the production of smokeless tobacco products, and previous studies suggest that TSNAs represent a much larger percentage of the total ‘toxicant load’ in smokeless products in comparison with cigarettes and other combustible tobacco products (Boffetta *et al*., 2008; Hecht, 2003). Decreasing the levels of NNN and NNK via the reduction of nitrate levels in dark tobaccos represents a promising strategy for lowering the levels of the two compounds that have been reported to be the most important toxicants in smokeless products.

Although TSNA formation has been particularly associated with the period of leaf cure (Bush *et al*., 2001), recent studies have also demonstrated that substantial TSNA production can occur during storage of the cured leaf. After 1 year of storage in a natural storage environment, Shi *et al*. (2013) observed total TSNA increases as high as 215% in sun‐cured and burley tobacco types. TSNA formation in this study was highly correlated with nitrate concentration. The addition of nitrate to flue‐cured tobaccos to a level comparable to that found in burley leaves lead to equivalent increases in storage‐associated TSNA formation between the two tobacco types. Even though TSNA formation after long‐term storage was not measured in our study, the results of Shi *et al*. (2013) strongly suggest that the low nitrate 35S:S523D‐NR tobacco plants described here would have a very low propensity for TSNA formation during storage.

The relative reduction in the levels of all TSNA species, except NNK, was even greater in the mainstream smoke of the low nitrate 35S:S523D‐NR cigarettes as compared to the corresponding unburned cut filler (Figure 5). The most straightforward explanation of these results would be that a certain proportion of the TSNAs found in the mainstream smoke are the products of pyrosynthesis, and that this process is minimized in cigarettes made with low nitrate tobaccos. Previous reports have differed in their conclusions regarding the degree with which TSNAs formed during combustion contribute towards the total TSNA pool found in the smoke. Early studies estimated that 50% or more of the NNN and NNK in mainstream smoke were synthesized during combustion (Adams *et al*., 1983; Hoffmann *et al*., 1977). Fischer *et al*. (1990), however, reached a very different conclusion, claiming that pyrosynthesis is negligible and that essentially all of the TSNAs found in mainstream smoke result from the transfer of those already existing in the filler. The results of our study are supportive of a model whereby some TSNAs in the smoke are products of combustion and that this process is dependent on the levels of available free nitrate. Although the amount of nitrate in a blended commercial cigarette would not be expected to be as high as that present in the current study using 100% burley tobacco, the incorporation low nitrate burley tobaccos in otherwise conventional cigarettes would likely serve to buffer against the propensity for TSNA formation during combustion.

Determining the metabolic fate of the N that is typically stored as nitrate in the 35S:S523D‐NR burley plants is also of interest. Although lowering the pools of stored nitrate in 35S:S523D‐NR plants was accompanied by substantial increases in ammonia and free amino acids in young growth chamber‐grown plants fertilized with exceptionally high levels of nitrate (19 mm), at more typical levels of N‐fertilization (8 mm) the effect on these downstream metabolites was relatively modest. Field‐grown plants accumulating ultra‐low nitrate levels not only displayed minimal changes in free amino acids, but also failed to show increases in alkaloid composition (Figure S6). Free amino acids, alkaloids and ammonia, however, only represent a subset of the N‐containing compounds within the cell. We are currently utilizing metabolomics and transcriptomics approaches to gain further insight into the metabolic and physiological consequences of plant cells expressing the S523D‐NR variant.

In conclusion, expression of a constitutively active S523D‐NR enzyme in burley tobacco plants resulted in dramatic reductions in the levels of free nitrate within the leaf. Reductions in nitrate were accompanied by significant decreases in the amounts of all TSNA species in the cured leaf and the associated mainstream smoke, including the documented animal carcinogens NNK and NNN. 35S:S523D‐NR‐mediated nitrate reduction in burley tobaccos has the potential for reducing TSNA synthesis at three distinct levels: (i) during the air‐curing process; (ii) during the period tobacco is being stored; and (iii) by mitigating formation that may occur as a result of pyrosynthesis. Further investigation will be needed to determine whether the low nitrate phenotype is associated with changes in agronomic performance (such as yield potential) or changes in other chemical constituents of the leaf and/or smoke beyond those measured here.

## Experimental procedures

### Plasmid constructs

The following genes were cloned using PCR: S523D‐NR—a tobacco *Nia2* cDNA containing a Ser to Asp substitution mutation introduced at codon 523 using site‐directed mutagenesis (Lillo *et al*., 2003); tr‐NR—a *Nia2* cDNA truncation mutant where codons 21–77 were removed by site‐directed mutagenesis (Nussaume *et al*., 1995); GS1: the full‐length cDNA of the tobacco *Gln1‐3* gene (Dubois *et al*., 1996); GOGAT: the full‐length cDNA of the *GLT1* gene of *Arabidopsis thaliana* (Lancien *et al*., 2002); and ICDH: a full‐length cDNA of a tobacco cytosolic isocitrate dehydrogenase (GenBank accession #X77944). Each cDNA was inserted individually into the binary vector pBI121 (Chen *et al*., 2003) by replacing the GUS reporter gene within this vector with the respective N‐assimilation pathway cDNA. This placed the constructs under the transcriptional control of the strong, constitutive CaMV 35S promoter. Plant vector pBI121 contains the *nptII* gene that enables selection of transformed cells using the antibiotic kanamycin. For constructs containing the *CYP82E4* (E4) promoter, the region of the vector containing the 35S promoter was excised and replaced with the 2.2 kb region immediately upstream of the *CYP82E4* start codon (Chakrabarti *et al*., 2008).

### Plant materials and field design

Burley breeding line DH98‐325‐6 #775 was transformed using the leaf disc method of Horsch *et al*. (1985). Several independent transgenic lines for each construct were assayed using semi‐quantitative RT‐PCR to identify the individual plants displaying the highest levels of transgene expression, relative to a tobacco actin gene. Green leaf tissue was used for the expression analysis of plants containing constructs under the transcriptional control of the CaMV 35S promoter. For plants containing the *CYP82*E4 promoter, expression analysis was conducted on leaves that were treated with ethephon as described by Chakrabarti *et al*. (2008). For each high expressing T0 individual, several T1 progeny were grown and genotyped using diagnostic primers for the cognate transgenes to distinguish progeny inheriting the transgene from null segregants. T1 progeny that assayed positive for possessing a given transgene were again assayed by semi‐quantitative RT‐PCR to test whether the high expression phenotype was faithfully transmitted to the next generation. Only lines that were shown to uniformly transmit the high expression phenotype in subsequent generations were used in this study.

Field evaluations of T2 generation plants were conducted in two locations (Rocky Mount, NC and Clayton, NC) using a randomized complete block design with individual plants as the experimental units. Agronomic practices typical for burley tobacco production, including topping, were employed at both locations.

### Growth conditions for controlled environmental chamber study

T2 transgenic tobacco plants and WT controls were germinated on a 2:1 peat:sand mix. Four‐week‐old seedlings were transplanted to individual cells and grown an additional 3 weeks at the North Carolina State University Phytotron facility using a standard nutrient solution and a 12 h 26 °C day/12 h 22 °C night regime. The 7‐week‐old plants were subsequently transplanted to 6″ pots filled with river sand, and for the next 7 days were watered with a nutrient solution containing excess nitrate (19 mm ${\mathrm{NO}}_{3}^{-}$) to allow them to adjust to transplant shock prior to initiation of the experiment. The 19 mm N nutrient solution consisted of: 5 mm Ca(NO_3_)_2_, 2 mm Mg(NO_3_)_2_, 5 mm KNO_3_, 1 mm KH_2_PO_4_, 0.5 mm K_2_SO_4_, 19 μm H_3_BO_3_, 3.7 μm MnCl_2_, 0.3 μm ZnSO_4_, 0.13 μm CuSO_4_, 0.05 μm Na_2_MoO_4_ and 10.0 μm 330 Fe‐Sequestrene. After the 7 day post‐transplant period, four to six plants from each genotypic group were watered either with a very low N (0.2 mm ${\mathrm{NO}}_{3}^{-}$), a medium N (8 mm ${\mathrm{NO}}_{3}^{-}$) or a high N (19 mm ${\mathrm{NO}}_{3}^{-}$) nutrient solution for 16 days. To maintain ionic and osmotic balance, ${\mathrm{SO}}_{4}^{2-}$ was substituted for ${\mathrm{NO}}_{3}^{-}$ across the low and medium solutions as appropriate (all micronutrients remained the same). Therefore, the low (0.2 mm ${\mathrm{NO}}_{3}^{-}$) N nutrient contained 0.1 mm Ca(NO_3_)_2_, 3 mm K_2_SO_4_, 2 mm MgSO_4_ and 4.9 mm CaSO_4_•2H_2_O (with no Mg(NO_3_)_2_ or KNO_3_) and the medium N (8 mm ${\mathrm{NO}}_{3}^{-}$) solution contained 4 mm Ca(NO_3_)_2_, 3 mm K_2_SO_4_, 2 mm MgSO_4_ and 1 mm CaSO_4_•2H_2_O (with no Mg(NO_3_)_2_ or KNO_3_).

### Phenotype analysis and processing

Biomass measurements were determined from the fresh weights of the aerial portion of the growth chamber‐grown plants. After weighing, the 4th or 5th leaf from the top was excised, its midrib removed, and the remaining lamina frozen at −80 °C and freeze‐dried for amino acid analysis. A small leaf sample (~300 mg) was also collected from a similar position from each plant for chlorophyll assays. The remaining leaf material was subsequently stripped from the main stem, placed in a paper bag and incubated in a drying oven at 65 °C for 2 days for nitrate, nitrite and ammonia analysis.

### Chlorophyll, nitrate, nitrite, ammonia and amino acid assays

Chlorophyll assays were conducted according to the protocol published in the *Protocol Exchange* (Ni *et al*., 2009) using a DU^®^ 640 Spectrophotometer (Beckman^™^ Coulter). Chlorophyll a, chlorophyll b and chlorophyll a + b concentrations were calculated according to the formulas in Arnon (1949). For the growth chamber experiments, dried leaf powder was assayed for nitrate and ammonia concentrations at the Environmental and Agricultural Testing Service laboratory at North Carolina State University according to the protocol in the Lachat Instruments (2008). For field‐grown samples, ground leaf tissue was analysed for nitrate at the University of Kentucky Tobacco Analytical Laboratory according to the protocol outlined in Crutchfield and Grove (2011). Nitrite analysis was conducted using the method described in Crutchfield and Burton (1989). Amino acid analysis was conducted on 100 mg of freeze‐dried leaf samples at the Biomanufacturing Training and Education Center at North Carolina State University (www.btec.ncsu.edu/services).

### Alkaloid and TSNA analysis of air‐cured leaves, cut filler and smoke

Nicotine, nornicotine, anatabine and anabasine levels in cured leaf samples were quantitated using a Perkin–Elmer Autosystem XL Gas Chromatograph according to previously established protocols (Jack *et al*., 2007). Total alkaloids were calculated as the sum of nicotine, nornicotine, anatabine and anabasine. Quantifications of NNN, NNK, NAT and NAB were conducted in accordance to ‘Method 1’ of Morgan *et al*. (2004). Total TSNAs represent the sum of NNN, NNK, NAT and NAB. Cut filler tobacco samples were extracted with a 100 mm ammonium acetate solution containing deuterated internal standards to enable quantification. Extracts were analysed by liquid chromatography tandem mass spectrometry (LC‐MS/MS) coupled with electrospray ionization (ESI). D4‐*N*‐nitrosonornicotine (D4‐NNN) was used as an internal standard for NNN, while D4‐4‐(methyl‐nitrosamine)‐1‐(3‐pyridyl)‐1‐butanone (D4‐NNK) served as an internal standard for NNK, NAT and NAB.

Test cigarettes were handmade, filled with an electric cigarette injector ‘Powermatic 2’ (http://powermatic2.com) using standard cigarette tubes. The weight of cut filler varied (averaging 800 mg) to get a resistance‐to‐draw corresponding to 120–140 mm water gauge. Cigarettes were subjected to machine smoking as outlined by Health Canada Method T‐115 (1999). Mainstream smoke was collected onto Cambridge filters and extracted with 100 mm ammonium acetate containing D4‐NNN and D4‐NNK standards. Extracts were analysed by LC‐MS/MS coupled with ESI and quantitated using D4‐NNN and D4‐NNK internal standards.

### Statistical analysis

The PROC GLM procedure of SAS 9.1 (SAS Institute, Cary, NC) was used to conduct an analysis of variance and to calculate means for each of the transgenic and WT lines included in this study. Because of heterogeneous variances, natural logarithmic data transformations were conducted to approximate normal distributions for the fresh weight, nitrate, ammonia, amino acid and TSNA measurements. Nontransformed values were used for all other datasets. Multiple comparisons of means were conducted according to the Ryan–Einot–Gabriel–Welsch multiple range test.

## Supporting information


**Figure S1** Individual T2 wild type (WT) and 35S:tr‐NR transgenic tobacco plants after watering with a 0.2 mm (bottom panel ‘L’), 8 mm (middle panel, ‘M’) or 19 mm (top panel, ‘H’) N nutrient solution for 16 days. Both genotypes are represented by six individuals (numbered 1 through 6) per N treatment level.
**Figure S2** Chlorophyll a (Ca, top left), chlorophyll b (Cb, top right) and chlorophyll a + b (Ca + b, bottom) contents of WT plants and 35S:tr‐NR, 35S:S523D‐NR, 35S:GS1, 35S:GOGAT and 35S:ICDH transgenic lines grown under three levels of N fertilization. Values shown represent the mean ± standard error of 4–6 plants for each genotype. Within each nitrate treatment level, means sharing the same letter are not significantly different from each other (*P *<* *0.05).
**Figure S3** Average ammonia content in leaves of WT plants and 35S:tr‐NR, 35S:S523D‐NR, 35S:GS1, 35S:GOGAT and 35S:ICDH transgenic lines grown under three levels of N fertilization. Values shown represent the nontransformed means ± standard errors of 4–6 plants for each genotype. Statistical tests were performed on transformed data (natural logarithmic transformation). Within each nitrate treatment level, means sharing the same letter are not significantly different from each other (*P *<* *0.05).
**Figure S4** Total free amino acid content in leaves of 35S:GOGAT, 35S:ICDH, 35S:tr‐NR, 35S:S523D‐NR, 35S:GS1 and WT plants grown under medium (8 mm) and high (19 mm) N fertilization. Values shown represent the mean ± standard error of 4–6 plants for each genotype. For each nitrate treatment level, means sharing the same letter are not significantly different from each other (*P *<* *0.05).
**Figure S5** Asn (top left), Gln (top right), Glu (bottom left) and Arg (bottom right) content in tobacco leaves of WT plants and 35S:tr‐NR, 35S:S523D‐NR, 35S:GS1, 35S:GOGAT and 35S:ICDH transgenic lines grown under medium (8 mm) and high (19 mm) N fertilization. Values shown represent the means ± standard errors of 4–6 plants for each genotype. Within each nitrate treatment level, means sharing the same letter are not significantly different from each other (*P *<* *0.05).
**Figure S6** Impact of 35S:S523D‐NR construct on the repartitioning of N from nitrate to select downstream N‐containing compounds. (a) Metabolic flow of nitrate N to amino acids and alkaloids. Arrows represent one or more enzymatic steps. (b) Heat map showing the fold increase or decrease in the accumulation of select metabolites in tobacco plants containing the 35S:S523D‐NR construct in comparison to WT controls. Young leaf samples were taken from plants grown in a controlled environmental chamber under conditions of medium (8 mm) or high (19 mm) nitrate fertilization. Cured leaf samples were from field grown plants. Orn, ornithine; n.d., not determined.

**Table S1** Mean fresh weight, chlorophyll, nitrate and ammonia measurements of plants expressing N‐assimilation pathway genes grown under three conditions of N fertilization
**Table S2** Amino acid concentration in plants expressing N‐assimilation pathway genes grown using medium (8 mm) and high (19 mm) N treatments
**Table S3** The effect of N‐assimilation pathway gene constructs on ${\mathrm{NO}}_{2}^{-}$ content (ppm of dry weight) in the leaves of T2 generation transgenic burley plants after 8 weeks of air‐curing
**Table S4** The effect of genotype on free amino acid concentration in cured burley tobacco leaves
