# Supplementary material for: Expression of a constitutively active nitrate reductase variant in tobacco reduces tobacco‐specific nitrosamine accumulation in cured leaves and cigarette smoke
Source: Plant Biotechnol J. 2016 Jan 23;14(7):1500–10. doi: 10.1111/pbi.12510 (PMC5066804; doi:10.1111/pbi.12510)
Supplement: Supplementary file 1 — Figure S1 Individual T2 wild type (WT) and 35S:tr‐NR transgenic tobacco plants after watering with a 0.2 mm (bottom panel ‘L’), 8 mm (middle panel, ‘M’) or 19 mm (top panel, ‘H’) N nutrient solution for 16 days. Both genotypes are represented by six individuals (numbered 1 through 6) per N treatment level. Figure S2 Chlorophyll a (Ca, top left), chlorophyll b (Cb, top right) and chlorophyll a + b (Ca + b, bottom) contents of WT plants and 35S:tr‐NR, 35S:S523D‐NR, 35S:GS1, 35S:GOGAT and 35S:ICDH transgenic lines grown under three levels of N fertilization. Values shown represent the mean ± standard error of 4–6 plants for each genotype. Within each nitrate treatment level, means sharing the same letter are not significantly different from each other (P < 0.05). Figure S3 Average ammonia content in leaves of WT plants and 35S:tr‐NR, 35S:S523D‐NR, 35S:GS1, 35S:GOGAT and 35S:ICDH transgenic lines grown under three levels of N fertilization. Values shown represent the nontransformed means ± standard errors of 4–6 plants for each genotype. Statistical tests were performed on transformed data (natural logarithmic transformation). Within each nitrate treatment level, means sharing the same letter are not significantly different from each other (P < 0.05). Figure S4 Total free amino acid content in leaves of 35S:GOGAT, 35S:ICDH, 35S:tr‐NR, 35S:S523D‐NR, 35S:GS1 and WT plants grown under medium (8 mm) and high (19 mm) N fertilization. Values shown represent the mean ± standard error of 4–6 plants for each genotype. For each nitrate treatment level, means sharing the same letter are not significantly different from each other (P < 0.05). Figure S5 Asn (top left), Gln (top right), Glu (bottom left) and Arg (bottom right) content in tobacco leaves of WT plants and 35S:tr‐NR, 35S:S523D‐NR, 35S:GS1, 35S:GOGAT and 35S:ICDH transgenic lines grown under medium (8 mm) and high (19 mm) N fertilization. Values shown represent the means ± standard errors of 4–6 plants for each genotype. [file PBI-14-1500-s002.docx]

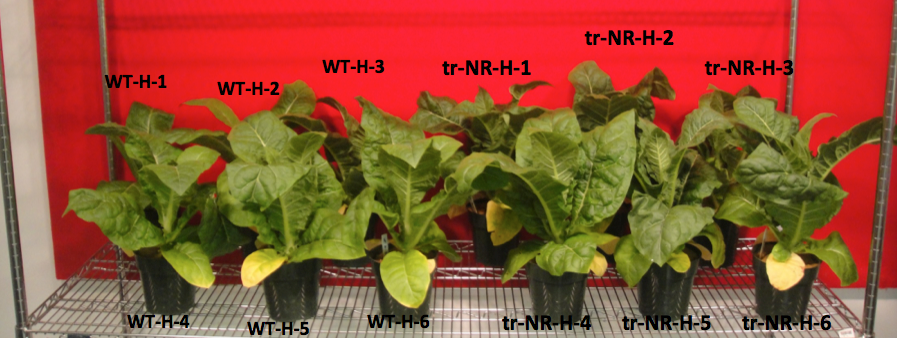

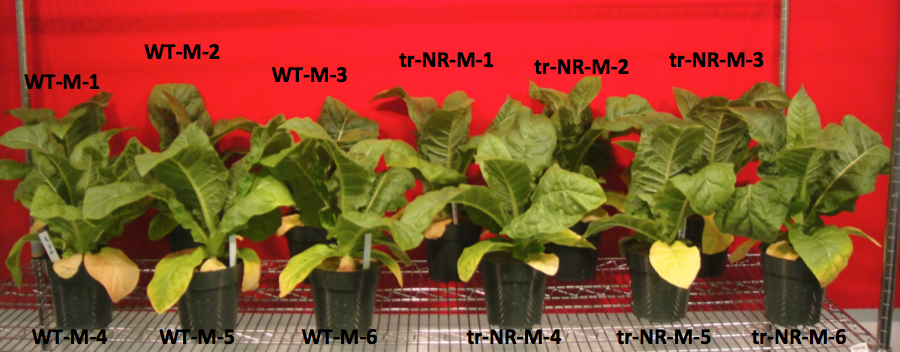

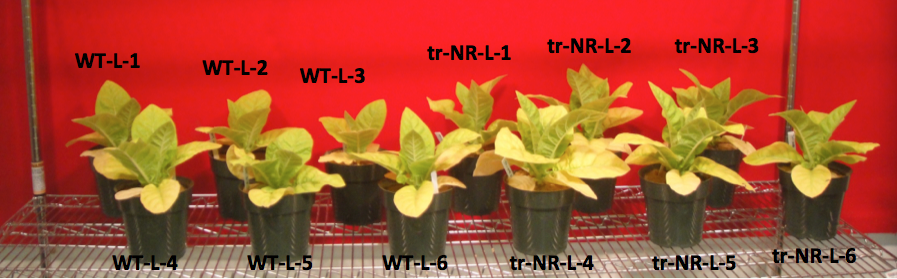


**Figure S1. Individual T2 wild type (WT) and 35S:tr-NR transgenic tobacco plants after watering with a 0.2 mM (bottom panel “L”), 8 mM (middle panel, “M”) or 19 mM (top panel, “H”) N nutrient solution for 16 days.** Both genotypes are represented by six individuals (numbered 1 through 6) per N treatment level.


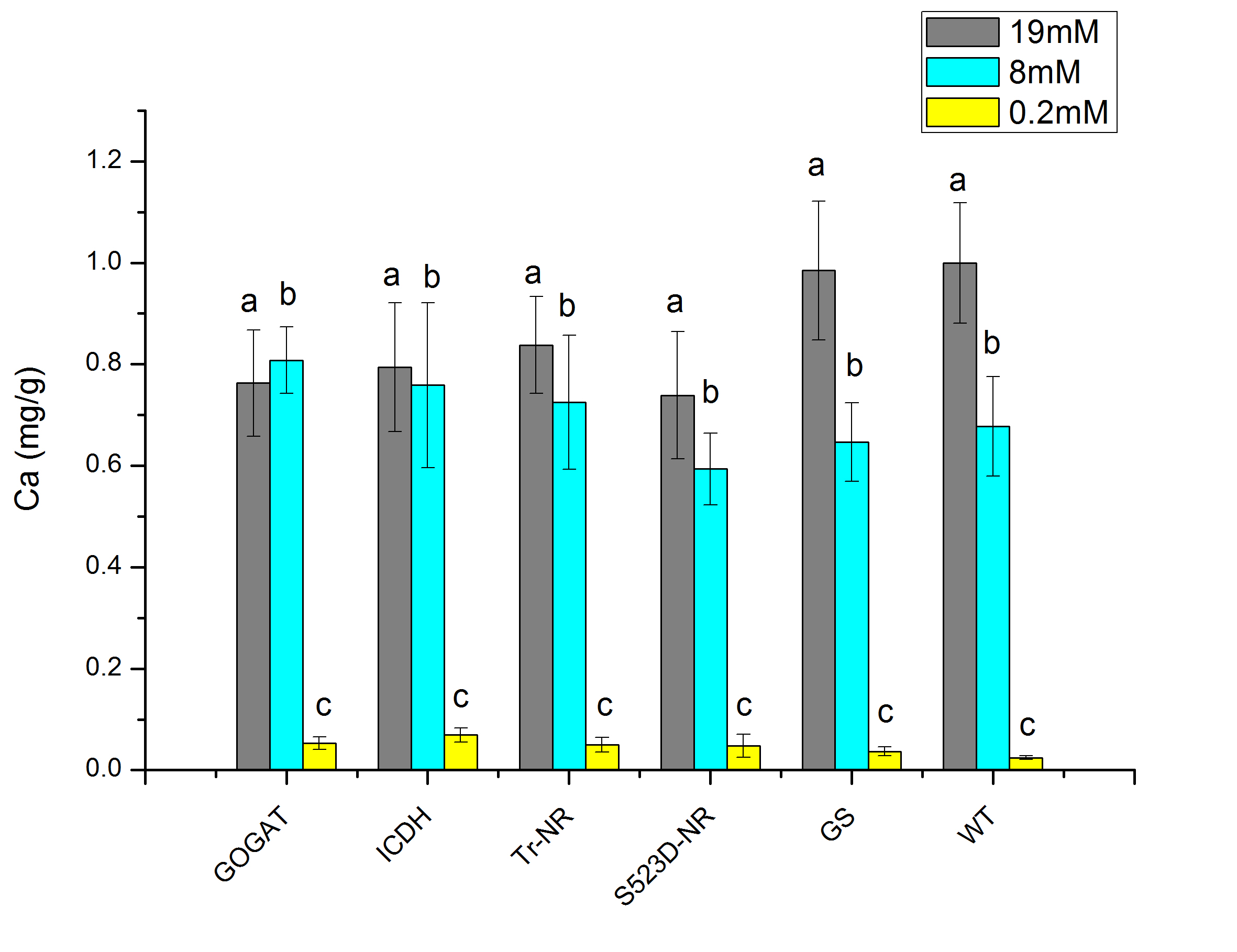

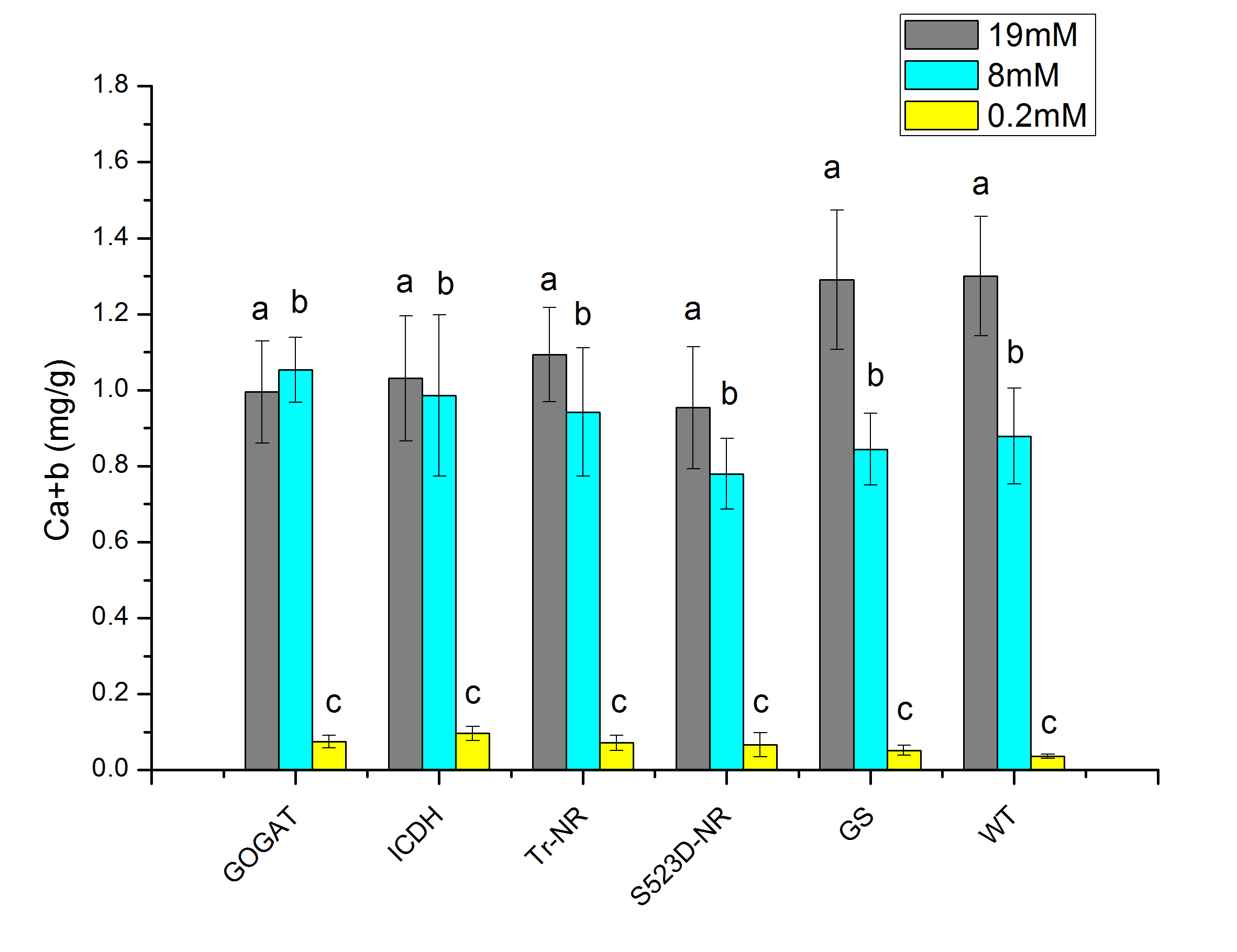

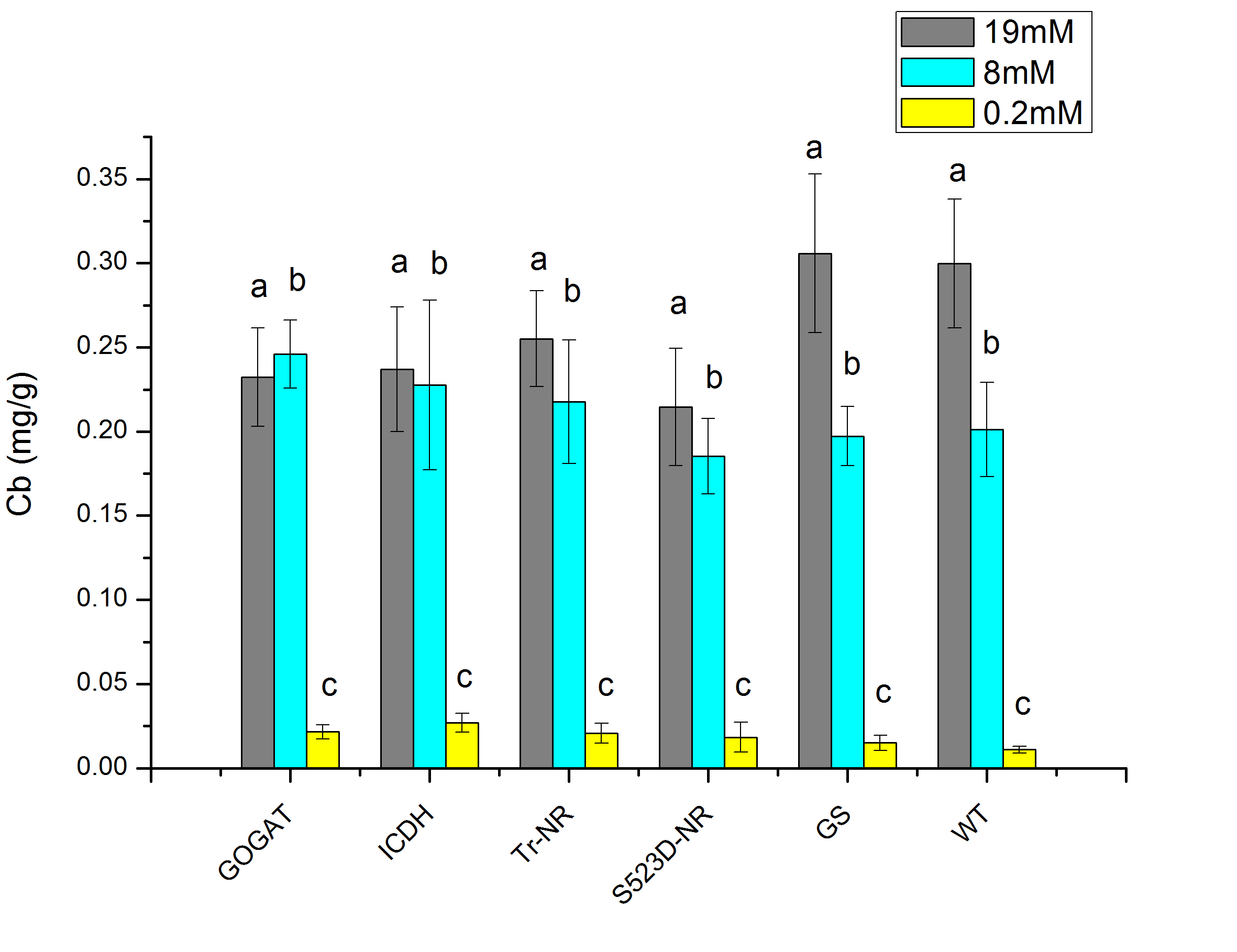


**Figure S2. Chlorophyll a (Ca, top left), chlorophyll b (Cb, top right) and chlorophyll a+b (Ca + b, bottom) contents of WT plants and 35S:tr-NR, 35S:S523D-NR, 35S:GS1, 35S:GOGAT and 35S:ICDH transgenic lines grown under three levels of N fertilization.** Values shown represent the mean ± standard error of 4-6 plants for each genotype. Within each nitrate treatment level, means sharing the same letter are not significantly different from each other (P < 0.05).


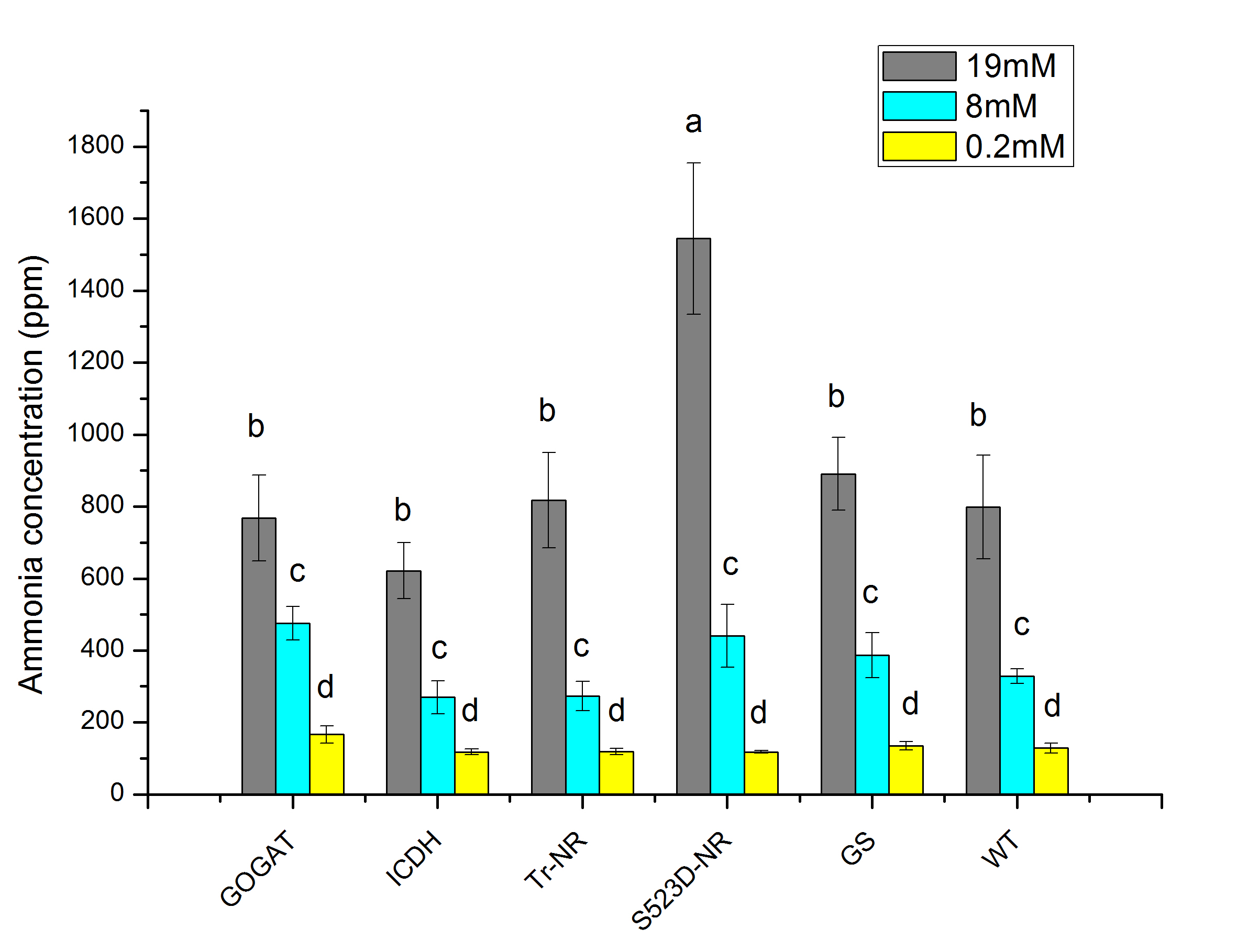


**Figure S3. Average ammonia content in leaves of WT plants and 35S:tr-NR, 35S:S523D-NR, 35S:GS1, 35S:GOGAT and 35S:ICDH transgenic lines grown under three levels of N fertilization.** Values shown represent the nontransformed means ± standard errors of 4-6 plants for each genotype. Statistical tests were performed on transformed data (natural logarithmic transformation). Within each nitrate treatment level, means sharing the same letter are not significantly different from each other (P < 0.05).


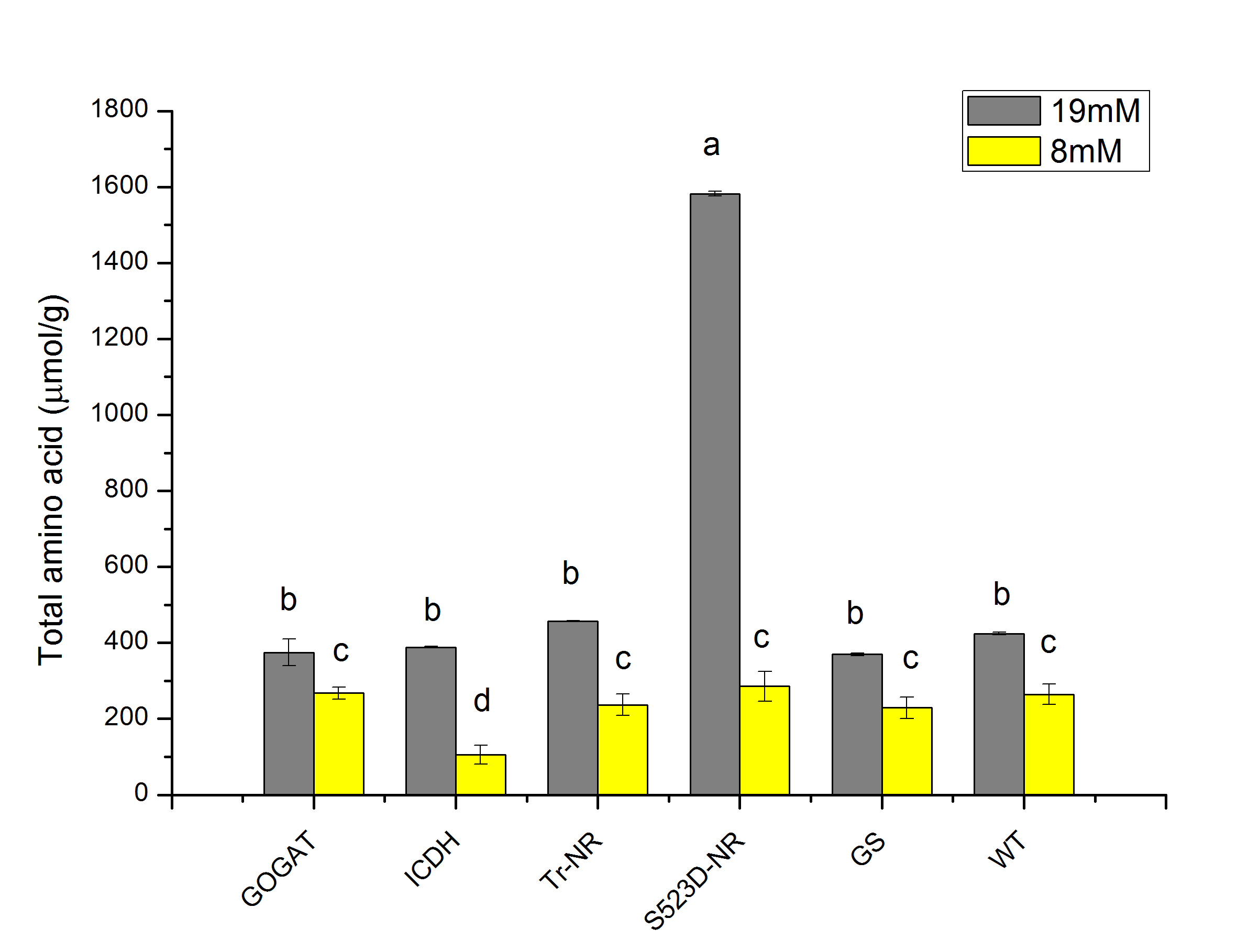


**Figure S4. Total free amino acid content in leaves of 35S:GOGAT, 35S:ICDH, 35S:tr-NR, 35S:S523D-NR, 35S:GS1 and WT plants grown under medium (8mM) and high (19mM) N fertilization.** Values shown represent the mean ± standard error of 4-6 plants for each genotype. For each nitrate treatment level, means sharing the same letter are not significantly different from each other (P < 0.05).


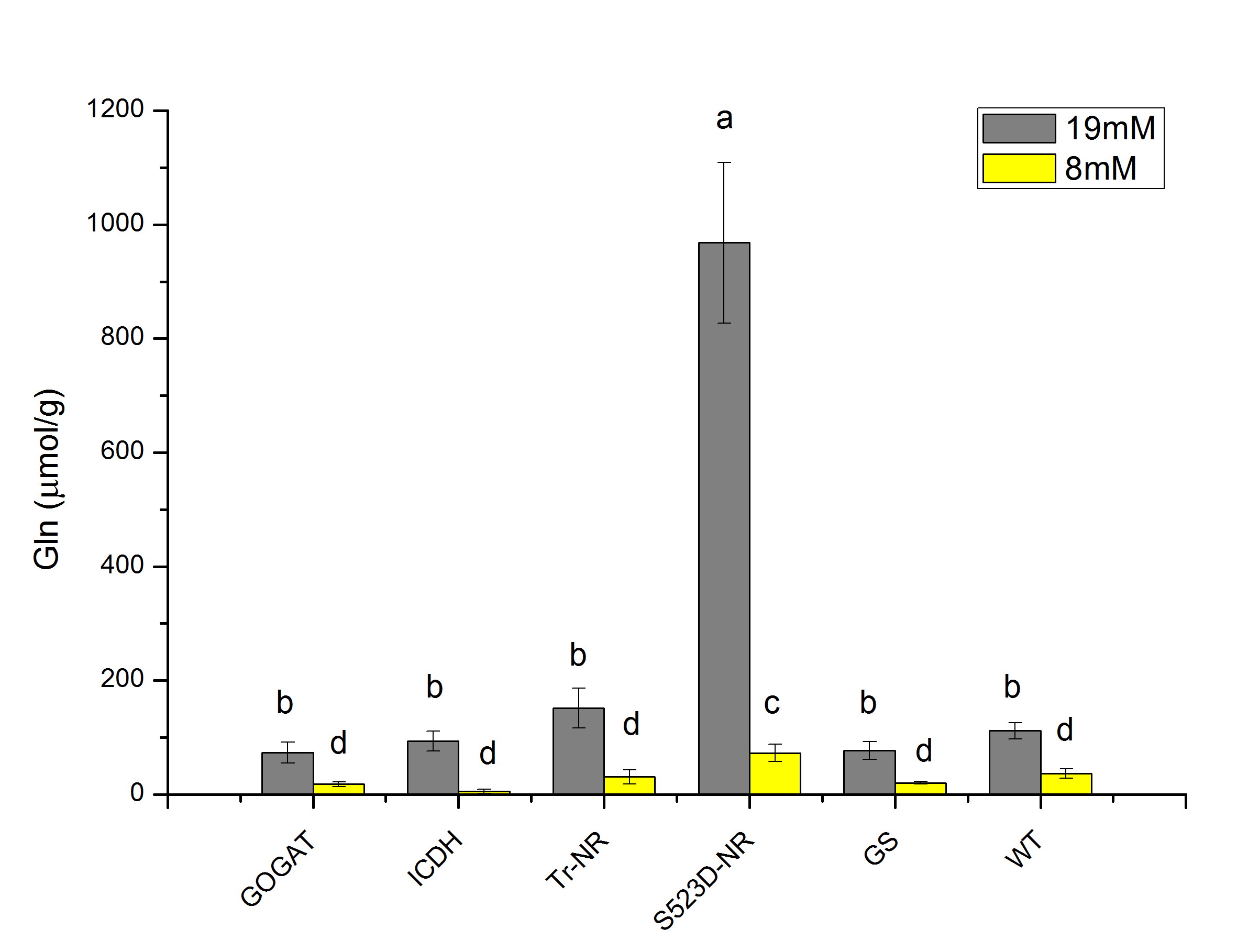


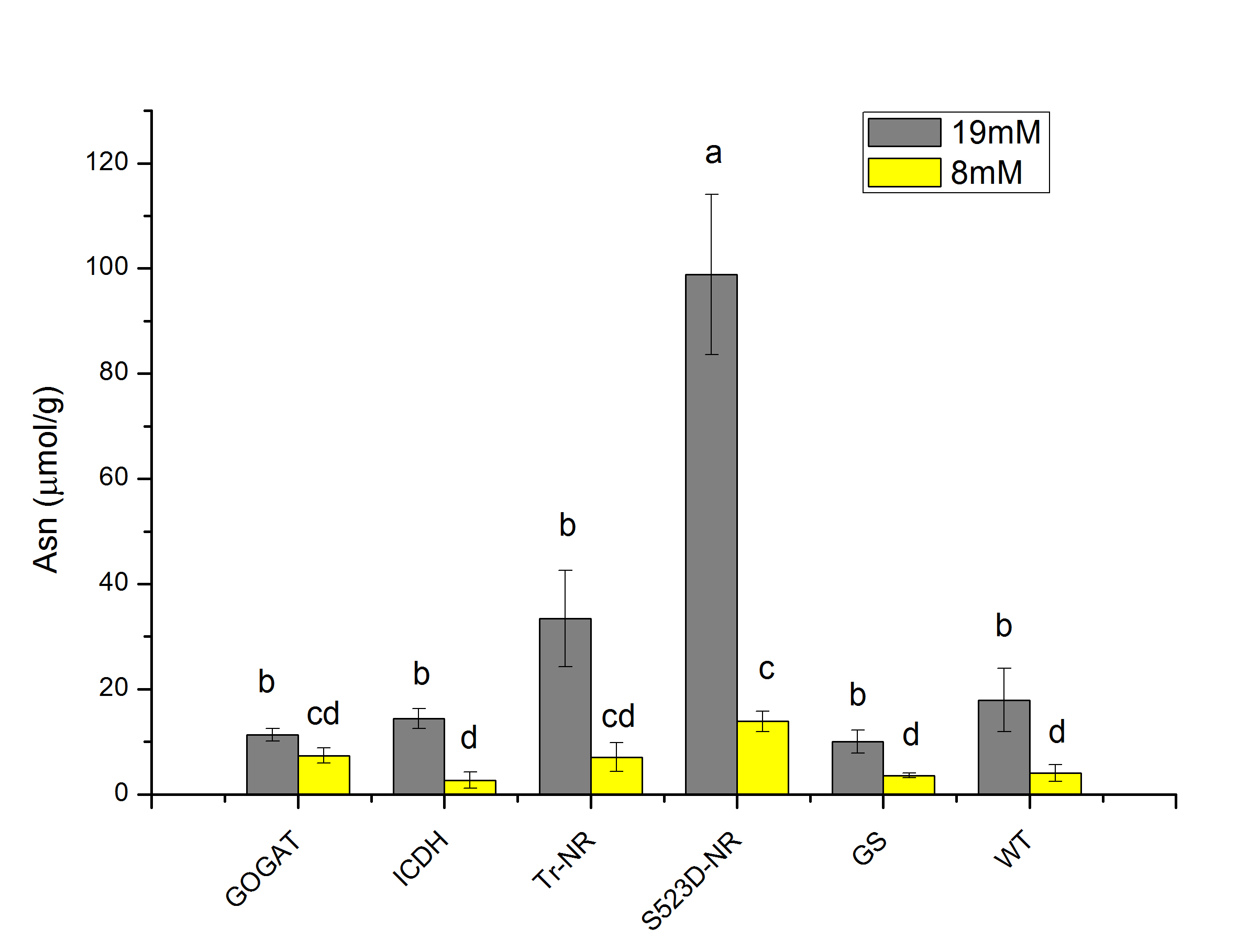


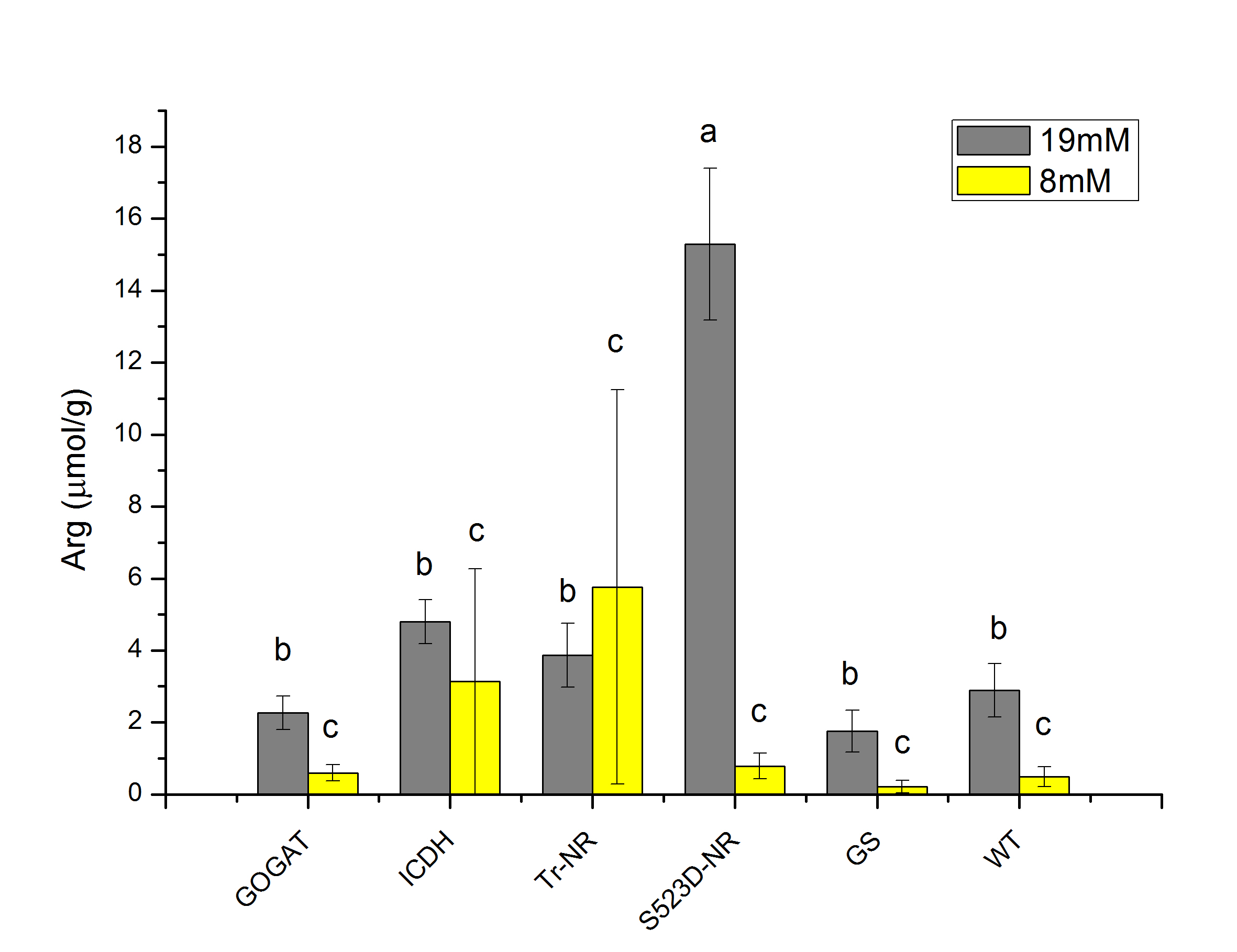

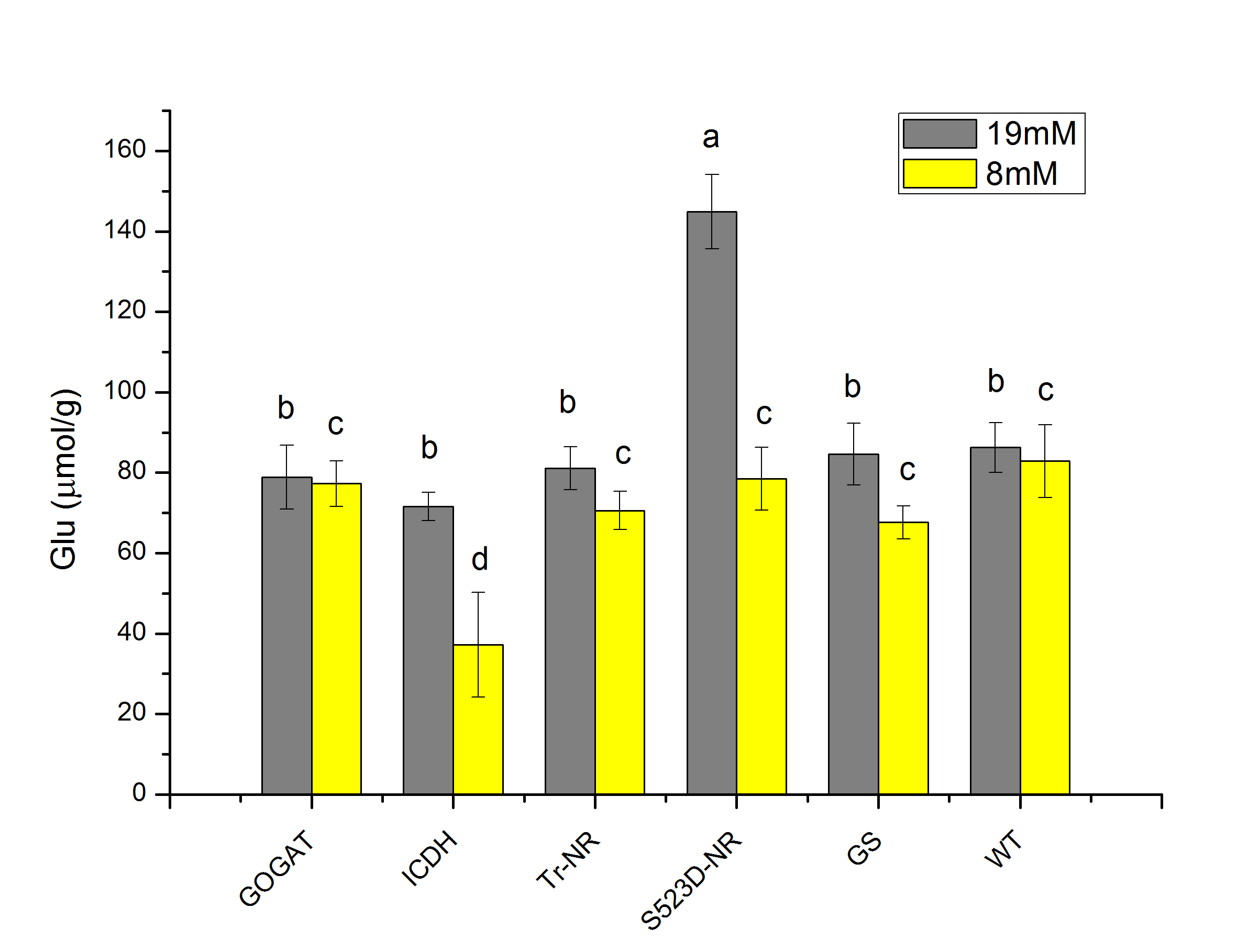


**Figure S5. Asn (top left), Gln (top right), Glu (bottom left) and Arg (bottom right) content in tobacco leaves of WT plants and 35S:tr-NR, 35S:S523D-NR, 35S:GS1, 35S:GOGAT and 35S:ICDH transgenic lines grown under medium (8mM) and high (19mM) N fertilization.** Values shown represent the means ± standard errors of 4-6 plants for each genotype. Within each nitrate treatment level, means sharing the same letter are not significantly different from each other (P < 0.05).

**Figure S6.**  **Impact of 35S:S523D-NR construct on the repartitioning of N from nitrate to select downstream N-containing compounds.** (A) Metabolic flow of nitrate N to amino acids and alkaloids. Arrows represent one or more enzymatic steps. (B) Heat map showing the fold increase or decrease in the accumulation of select metabolites in tobacco plants containing the 35S:S523D-NR construct in comparison to WT controls. Young leaf samples were taken from plants grown in a controlled environmental chamber under conditions of medium (8 mM) or high (19 mM) nitrate fertilization. Cured leaf samples were from field grown plants. Orn, ornithine; n.d., not determined.
