# Supplementary material for: Expression of a constitutively active nitrate reductase variant in tobacco reduces tobacco‐specific nitrosamine accumulation in cured leaves and cigarette smoke
Source: Plant Biotechnol J. 2016 Jan 23;14(7):1500–10. doi: 10.1111/pbi.12510 (PMC5066804; doi:10.1111/pbi.12510)
Supplement: Supplementary file 2 — Table S1 Mean fresh weight, chlorophyll, nitrate and ammonia measurements of plants expressing N‐assimilation pathway genes grown under three conditions of N fertilization Table S2 Amino acid concentration in plants expressing N‐assimilation pathway genes grown using medium (8 mm) and high (19 mm) N treatments Table S3 The effect of N‐assimilation pathway gene constructs on NO2− content (ppm of dry weight) in the leaves of T2 generation transgenic burley plants after 8 weeks of air‐curing Table S4 The effect of genotype on free amino acid concentration in cured burley tobacco leaves [file PBI-14-1500-s001.docx]

**Table S1. Mean fresh weight, chlorophyll, nitrate and ammonia measurements of plants expressing N-assimilation pathway genes grown under three conditions of N fertilization.**

| Transgenic construct | N Treatment | Fresh weight (g) | Chl a (mg/g) | Chl b (mg/g) | Chl a+b (mg/g) | Nitrate Conc (ppm) | Ammonia Conc (ppm) |
| --- | --- | --- | --- | --- | --- | --- | --- |
| 35S:GOGAT (*n*=6)  35S:GOGAT (*n*=6)  35S:GOGAT (*n*=6) | 0.2mM | 28.420 | 0.053 | 0.022 | 0.075 | 99.100 | 166.692 |
|  | 8mM | 107.688 | 0.808 | 0.246 | 1.053 | 1923.283 | 475.642 |
|  | 19mM | 132.798 | 0.763 | 0.232 | 0.995 | 15643.513 | 767.839 |
| 35S:ICDH (*n*=5) | 0.2mM | 30.796 | 0.069 | 0.027 | 0.096 | 67.085 | 118.261 |
| 35S:ICDH (*n*=4) | 8mM | 106.528 | 0.759 | 0.228 | 0.986 | 2257.586 | 269.833 |
| 35S:ICDH (*n*=5) | 19mM | 139.472 | 0.794 | 0.237 | 1.031 | 10118.411 | 621.612 |
| 35S:tr-NR (*n*=6) | 0.2mM | 43.270 | 0.050 | 0.021 | 0.071 | 59.426 | 119.206 |
| 35S:tr-NR (*n*=6) | 8mM | 127.707 | 0.725 | 0.218 | 0.942 | 846.754 | 273.316 |
| 35S:tr-NR (*n*=6) | 19mM | 160.602 | 0.838 | 0.255 | 1.093 | 8953.107 | 817.139 |
| 35S:S523D-NR (*n*=5) | 0.2mM | 38.548 | 0.048 | 0.018 | 0.066 | 59.917 | 117.472 |
| 35S:S523D-NR (*n*=5) | 8mM | 114.420 | 0.594 | 0.185 | 0.779 | 124.627 | 440.455 |
| 35S:S523D-NR (*n*=6) | 19mM | 120.892 | 0.739 | 0.215 | 0.954 | 2026.337 | 1544.655 |
| 35S:GS1 (*n*=6) | 0.2mM | 34.953 | 0.037 | 0.015 | 0.052 | 79.219 | 134.696 |
| 35S:GS1 (*n*=6) | 8mM | 113.643 | 0.647 | 0.197 | 0.844 | 2391.705 | 386.519 |
| 35S:GS1 (*n*=5) | 19mM | 127.266 | 0.985 | 0.306 | 1.290 | 12614.557 | 890.905 |
| Wild type (*n*=6) | 0.2mM | 28.673 | 0.024 | 0.011 | 0.035 | 75.771 | 128.621 |
| Wild type (*n*=6) | 8mM | 110.357 | 0.678 | 0.201 | 0.879 | 2295.164 | 328.894 |
| Wild type (*n*=6) | 19mM | 130.175 | 1.000 | 0.300 | 1.300 | 14918.953 | 798.592 |

**Table S2. Amino acid concentration in plants expressing N-assimilation pathway genes grown using medium (8mM) and high (19mM) N treatments.**

| Metabolite concentration (μmol/g dry weight) | | | | | | |
| --- | --- | --- | --- | --- | --- | --- |
|  | WT | | 35S:tr-NR | | 35S:S523D-NR | |
|  | 8mM | 19mM | 8mM | 19mM | 8mM | 19mM |
| His | 0.916 | 1.670 | 0.934 | 1.830 | 1.450 | 7.584 |
| Asn | 4.057 | 17.934 | 7.079 | 33.443 | 13.880 | 98.851 |
| Ser | 22.750 | 33.695 | 18.203 | 27.970 | 17.308 | 46.370 |
| Gln | 36.651 | 111.772 | 30.583 | 151.395 | 72.416 | 968.148 |
| Arg | 0.490 | 2.898 | 5.767 | 3.869 | 0.791 | 15.292 |
| Gly | 4.461 | 27.494 | 2.832 | 11.812 | 1.856 | 48.442 |
| Asp | 18.533 | 26.219 | 10.239 | 17.290 | 15.605 | 44.033 |
| Glu | 82.841 | 86.255 | 70.556 | 81.072 | 78.470 | 144.897 |
| Thr | 36.234 | 28.762 | 24.079 | 30.702 | 22.548 | 41.732 |
| Ala | 11.124 | 28.160 | 16.904 | 23.089 | 10.784 | 40.634 |
| Pro | 15.916 | 20.291 | 20.839 | 31.498 | 22.776 | 58.392 |
| Cys | 0.041 | 0.154 | 0.135 | 0.096 | 0.077 | 0.090 |
| Lys | 1.522 | 1.696 | 1.613 | 1.925 | 1.686 | 3.295 |
| Tyr | 3.774 | 5.830 | 3.697 | 6.429 | 3.486 | 6.773 |
| Met | 0.224 | 0.720 | 0.537 | 0.492 | 0.601 | 1.447 |
| Val | 12.749 | 16.961 | 12.747 | 19.509 | 13.227 | 31.370 |
| Ile | 1.549 | 2.367 | 1.450 | 2.192 | 1.395 | 4.594 |
| Leu | 4.088 | 3.705 | 2.966 | 4.562 | 3.053 | 7.697 |
| Phe | 4.700 | 6.572 | 4.047 | 5.461 | 3.070 | 8.943 |
| Trp | 1.377 | 1.422 | 1.628 | 1.787 | 1.429 | 3.121 |
| Total | 263.998 | 424.578 | 236.835 | 456.424 | 285.909 | 1581.703 |

**Table S2 continued**

| Metabolite concentration (μmol/g dry weight) | | | | | | |
| --- | --- | --- | --- | --- | --- | --- |
|  | 35S:GS1 | | 35S:ICDH | | 35S:GOGAT | |
|  | 8mM | 19mM | 8mM | 19mM | 8mM | 19mM |
| His | 1.024 | 1.091 | 1.060 | 1.605 | 1.013 | 1.713 |
| Asn | 3.599 | 10.052 | 2.715 | 14.428 | 7.400 | 11.330 |
| Ser | 17.576 | 28.809 | 5.631 | 32.661 | 22.804 | 30.923 |
| Gln | 20.609 | 77.103 | 5.325 | 93.425 | 17.624 | 73.545 |
| Arg | 0.215 | 1.758 | 3.134 | 4.797 | 0.598 | 2.266 |
| Gly | 1.731 | 23.584 | 0.441 | 32.613 | 1.761 | 19.006 |
| Asp | 12.647 | 25.376 | 6.358 | 18.297 | 15.992 | 27.733 |
| Glu | 67.637 | 84.602 | 37.214 | 71.596 | 77.295 | 78.855 |
| Thr | 55.242 | 36.986 | 13.128 | 28.333 | 51.094 | 40.043 |
| Ala | 10.023 | 21.379 | 6.448 | 25.789 | 22.996 | 22.634 |
| Pro | 13.612 | 18.443 | 8.120 | 29.038 | 16.345 | 26.077 |
| Cys | 0.145 | 0.110 | 0.148 | 0.302 | 0.115 | 0.055 |
| Lys | 1.650 | 1.850 | 1.819 | 2.157 | 1.944 | 2.033 |
| Tyr | 2.705 | 4.905 | 2.069 | 4.616 | 3.643 | 4.683 |
| Met | 0.635 | 0.730 | 0.332 | 0.649 | 0.515 | 0.567 |
| Val | 9.064 | 18.887 | 6.092 | 13.562 | 13.749 | 16.397 |
| Ile | 1.701 | 2.148 | 1.038 | 2.601 | 2.179 | 3.928 |
| Leu | 4.558 | 4.642 | 2.006 | 3.645 | 4.008 | 3.902 |
| Phe | 3.718 | 5.718 | 1.964 | 6.798 | 4.606 | 6.093 |
| Trp | 1.411 | 1.515 | 0.620 | 1.677 | 1.610 | 2.687 |
| Total | 229.501 | 369.688 | 105.663 | 388.590 | 267.292 | 374.471 |

| **Table S3.  The effect of N-assimilation pathway gene constructs on NO_2_^-^ content (ppm of dry weight) in the leaves of T2 generation transgenic burley plants after eight weeks of air-curing.** | | | |
| --- | --- | --- | --- |
|  | | | |
| **REGWQ Grouping** | **Mean** | ***n*** | **Genotype** |
| A | 2.4403 | 63 | E4:GOGAT |
| BA | 2.3559 | 79 | WT |
| BA | 2.3236 | 75 | E4:S523D-NR |
| BA | 2.3052 | 79 | 35S:GOGAT |
| BA | 2.2790 | 76 | E4:tr-NR |
| BA | 2.1125 | 70 | 35S:tr-NR |
| B | 1.9574 | 60 | 35S:S523D-NR |

*Means with the same letter are not significantly different at α=0.05*.

| **Table S4.** **The effect of genotype on free amino acid concentration in cured burley tobacco leaves.** | | | |
| --- | --- | --- | --- |
|  | **35S:S523D-NR**^b^ | **35S:GOGAT**^b^ | **WT**^b^ |
| Ala | 1729^a,c^ | 641 | 628 |
|  | A^d^ | B | B |
|  |  |  |  |
| Arg | 461 | 487 | 554 |
|  | A | A | A |
|  |  |  |  |
| Asn | 11208 | 7461 | 7647 |
|  | A | A | A |
|  |  |  |  |
| Asp | 14593 | 9652 | 7927 |
|  | A | B | B |
|  |  |  |  |
| Cys | 18 | 14 | 27 |
|  | A | A | A |
|  |  |  |  |
| Gln | 1521 | 1312 | 1337 |
|  | A | A | A |
|  |  |  |  |
| Glu | 2144 | 959 | 1342 |
|  | A | C | B |
|  |  |  |  |
| Gly | 742 | 590 | 552 |
|  | A | AB | B |
|  |  |  |  |
| His | 712 | 561 | 626 |
|  | A | A | A |
|  |  |  |  |
| Ile | 159 | 113 | 128 |
|  | A | A | A |
|  |  |  |  |
| Leu | 238 | 192 | 199 |
|  | A | A | A |
|  |  |  |  |
| Lys | 464 | 440 | 502 |
|  | A | A | A |
|  |  |  |  |
| Met | 120 | 106 | 129 |
|  | A | A | A |
| **Table S4. continued** | | | |
|  | **35S:S523D-NR** | **35S:GOGAT** | **WT** |
| Phe | 1409 | 1247 | 1121 |
|  | A | A | A |
|  |  |  |  |
| Pro | 276 | 178 | 197 |
|  | A | A | A |
|  |  |  |  |
| Ser | 735 | 678 | 474 |
|  | A | A | A |
|  |  |  |  |
| Thr | 609 | 429 | 397 |
|  | A | B | B |
|  |  |  |  |
| Trp | 1439 | 1293 | 1456 |
|  | A | A | A |
|  |  |  |  |
| Tyr | 154 | 167 | 173 |
|  | A | A | A |
|  |  |  |  |
| Val | 392 | 369 | 344 |
|  | A | A | A |
|  |  |  |  |
| Total amino acids | 66890 | 52007 | 53676 |
|  | A | A | A |
|  |  |  |  |
| NH_3_ | 30180 | 27718 | 28748 |
|  | A | A | A |
| *a.  Free amino acid concentrations are in nmol per gram dry matter.* | | | |
| *b. n=33 for each genotype.* | | | |
| *c. Statistical differences were analyzed using the natural logarithms of the concentration data; the data shown in the table represent arithmetic means.* | | | |
| *d. Means with the same letter are not significantly different at alpha=0.05 according to REGWG grouping.* | | | |
|  | | | |
